# MOF-74(M) (M = Mg(II), Fe(II), Ni(II)) frameworks to enable accelerated redox kinetics for Li–S batteries

**DOI:** 10.1038/s41598-025-22340-4

**Published:** 2025-11-03

**Authors:** D. Capková, T. Kazda, N. Király, D. Volavka, P. Obšatník, A. Šimek, P. Čudek, D. Matoga, J. Bednarčík, A. Straková Fedorková, V. Kuchárová, V. Hornebecq, K. M. Ryan, M. Almáši

**Affiliations:** 1 Department of Chemical Sciences, Bernal Institute, University of Limerick, Limerick, V94 T9PX Ireland; 2 Department of Electrical and Electronic Technology, Faculty of Electrical Engineering and Communication, Brno University of Technology, Technická 10, 616 00 Brno, Czech Republic; 3 Department of Inorganic Chemistry, Faculty of Sciences, Pavol Jozef Šafárik University in Košice, Moyzesova 11, 040 01 Kosice, Slovak Republic; 4 Department of Solid State Physics, Faculty of Sciences, Pavol Jozef Šafárik University in Košice, Park Angelinum 9, 040 01 Kosice, Slovak Republic; 5 Faculty of Chemistry, Jagiellonian University, Gronostajowa 2, 30-387 Kraków, Poland; 6 Institute of Experimental Physics, Slovak Academy of Sciences, Watsonova 47, 040 01 Kosice, Slovak Republic; 7 Department of Physical Chemistry, Faculty of Sciences, Pavol Jozef Šafárik University in Košice, Moyzesova 11, 040 01 Kosice, Slovak Republic; 8 CNRS, MADIREL, Aix-Marseille University, 133 97 Marseille, France

**Keywords:** Lithium–sulphur batteries, Sulphur cathode, Metal–organic framework, MOF-74, Energy storage, Chemistry, Energy science and technology, Materials science

## Abstract

This study presents the development of a composite electrode material for lithium–sulphur (Li–S) batteries, combining MOF-74 with carbon black and sulphur. The MOF-74 structures, incorporating Ni(II), Mg(II), and Fe(II) metal ions, were synthesized via a solvothermal method and used to encapsulate sulphur. The microporous nature of MOF-74 facilitates the physical confinement and storage of sulphur, potentially enhancing the performance of Li–S batteries. The investigation focuses on how different central metal ions in MOF-74 influence the performance of sulphur-based electrodes. Among the metal ions studied, Fe(II) and Mg(II) were selected for their low toxicity, cost-effectiveness, and availability, while Ni(II) was included for its high catalytic properties. The materials were thoroughly characterized using infrared spectroscopy, thermogravimetric analysis, scanning electron microscopy, powder X-ray diffraction, elemental analysis and X-ray photoelectron spectroscopy. The thermal stability and textural properties of the materials were assessed, showing that MOF-74(Mg) exhibited the highest stability, followed by MOF-74(Ni) and MOF-74(Fe). Nitrogen adsorption/desorption measurements indicated that the specific surface area and pore volume varied with activation temperature, impacting the material’s performance. Among the tested materials, MOF-74(Ni) exhibited the strongest interaction with sulfur, as confirmed by XPS analysis. Electrochemical tests revealed that the S/MOF-74(Ni) electrode demonstrated superior stability and capacity retention with a minimal capacity fading rate of 0.001% per cycle over 200 cycles, achieving a reversible capacity of 465 mAh g^−1^ and a capacity retention of 99.75%. In contrast, the S/MOF-74(Fe) electrode showed significantly reduced performance. A structure–performance correlation was established to assess sulfur interaction, electrode stability, and degradation behavior. Overall, the results highlight that MOF-74(Ni) offers the most promising performance due to its effective sulphur immobilization and superior electrochemical properties compared to MOF-74(Mg) and MOF-74(Fe).

**Supplementary Information:**

The online version contains supplementary material available at 10.1038/s41598-025-22340-4.

## Introduction

Energy storage technology is essential for the sustainable improvement of human society, especially in the present period when various electric vehicles are coming to the fore. Lithium-ion (Li-ion) batteries have been in increasing demand in recent decades; however, their energy density is reaching an upper limit, pushing researchers to look for next-generation alternatives^1,2^. Considering the high abundance on the Earth’s crust, low cost, environmental friendliness, high theoretical capacity (1675 mAh g^−1^) and energy density (2600 Wh kg^−1^), lithium-sulphur (Li–S) batteries are a promising alternative for next-generation batteries^3–6^. However, its commercial production is hampered by a series of challenging drawbacks. The capacity of Li–S batteries depends on the redox reaction of sulphur during the charging and discharging processes, leading to the production of large amounts of polysulphides^7^, and their dissolution leads to the shuttle effect^8^. Other drawbacks are the volumetric expansion of sulphur (~ 30%), poor electronic conductivity of sulphur and discharge product (Li_2_S), and low utilization of active material^9^. These issues cause unsatisfactory cycle performance, rapid capacity loss, and a reduction in the amount of active material^10^.

Different solutions have been proposed in terms of cathode materials to overcome these issues. Porous carbon materials are preferred due to their high conductivity, such as carbon nanotubes^11^, microporous^12^/mesoporous^13^/macroporous carbons^14^, and graphene^15^. Carbon materials can also be used as substrates for compounding other materials to improve the electrochemical performance of the electrode material^16^. However, due to the weak van der Waals interaction, non-polar carbonaceous materials can only mitigate the polysulfide shift to a certain extent. Consequently, chemical confinement has been proposed to remove the weaknesses of carbonaceous materials. Therefore, various polar materials such as metal oxides^17^, heteroatom doped-carbon^18^ and polymers^19^ have been studied to capture polysulphides and mitigate their shuttling. Nevertheless, the effect of this strategy is still unsatisfactory under conditions of high sulphur loading and lean electrolyte due to the adsorption sites of polar materials being finite and the slow conversion kinetics of polysulphides being unchanged. In this regard, the research attention shifts from physical limitations by carbon materials to chemical entrapments using polar materials, which may improve the kinetics of polysulphide conversion via a catalytic effect^20^. The key to improving the overall performance of Li–S batteries is the design of catalytic materials that synchronously combine efficient polysulphide binding and fast conversion along with smooth adsorption, diffusion and conversion of polysulphides.

A class of porous coordination polymers called metal–organic frameworks (MOFs), which have a large surface area, adjustable pore size and a unique porous structure, have proven to be effective catalysts for Li–S batteries^21^. They can facilitate the transfer of electrons and ions, reduce the activation energy and help to maintain the structural integrity which can lead to speeding up the reactions, improving the battery’s performance and efficiency. MOFs are hybrid inorganic–organic materials consisting of metal ions or clusters bridged by organic linkers to form robust frameworks with permanent porosity^22^. Due to their unique structure, they find applications in many research areas, such as drug delivery^23,24^, gas adsorption and separation^25–27^, heterogeneous catalysis^28,29^, ion exchange^30,31^, sensing^32–34^ or as additives in cathode materials for Li–S batteries^35,36^. Zeolitic imidazolate frameworks (ZIFs) are among the most widely used MOFs in Li–S batteries. Zhou et al. fabricated a sulphur cathode containing carbonized ZIF-67, which exhibited a discharge capacity of 702 mAh g^−1^ at 0.2 C^37^. Yang et al. described ZIF-8 deposited on carbon cloth as a sulphur host; the capacity of 1036 mAh g^−1^ at 0.2 C was achieved^38^. MOF-74, which has a high number of active sites (coordinatively unsaturated sites (CUSs)), is commonly used as a catalyst in Li-air batteries^39,40^. Furthermore, using density functional theory (DFT) calculations, it was confirmed that MOF-74 can chemically adsorb polysulphides to the active sites^41^. The MOF-74 showed a good performance in solid-state Li-ion^42^ or as an interlayer in Li–S^43^. The high number of active sites in synergy with the catalytic effect of metal could effectively enhance the performance of Li–S batteries.

**Fig. 1:**
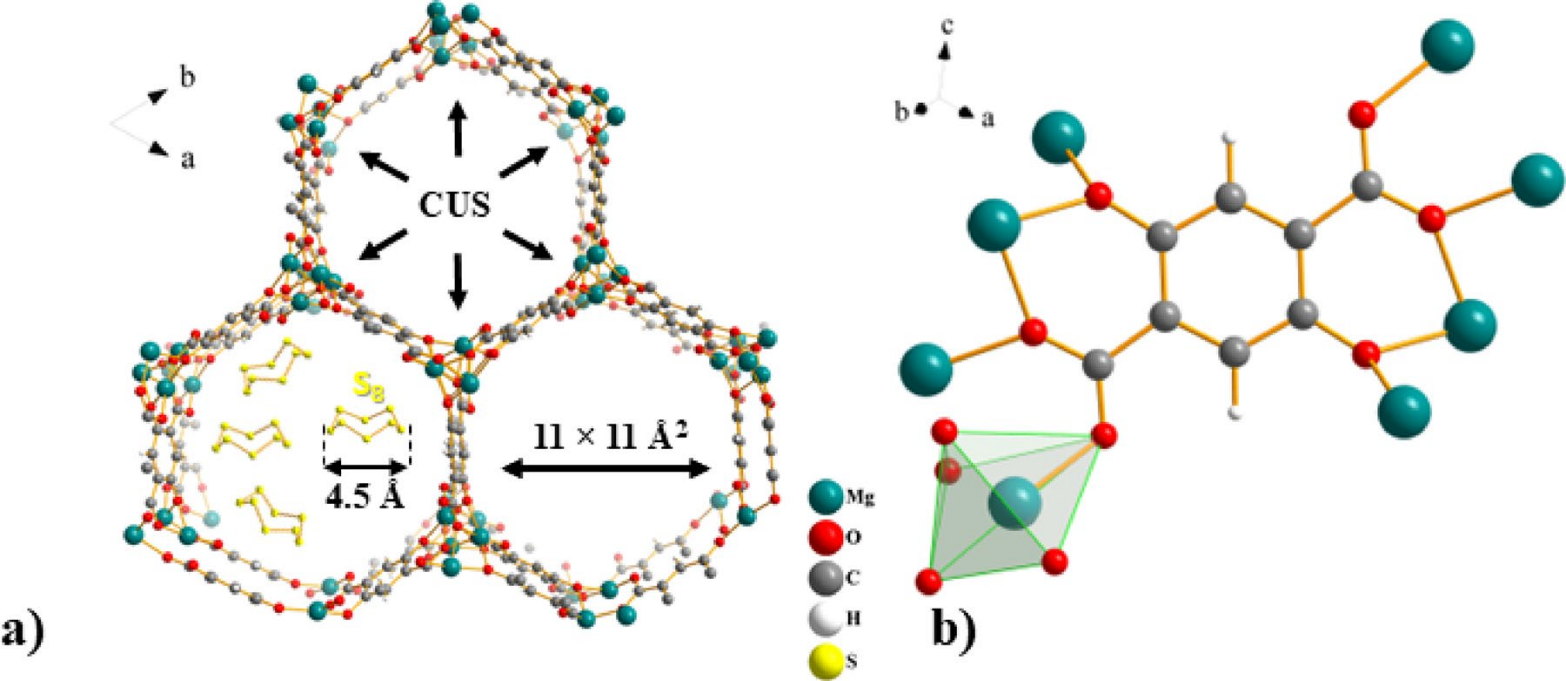
(**a**) A view of the MOF-74 dehydrated framework along the *c* crystallographic axis showing the honeycomb topology containing one-dimensional hexagonal channels lined with coordinatively unsaturated sites (CUSs) and comparison of pore size (11 Å) and S_8_ molecule (4.5 Å)^56^. (**b**) Coordination mode of DOBDC^4−^ ligand and coordination polyhedron around central atom present in MOF-74^57^.

The frameworks of MOF-74(M) (where M represents a divalent metal cation) display an isostructural topology with the formula {[M_2_(DOBDC)(H_2_O)_2_]·G}_n_ (DOBDC^4−^ = 2,5-dioxido-1,4-benzene-dicarboxylate, G = guest molecules), where the metal entities serve as nodes linked by organic linkers to form honeycomb skeleton containing hexagonal pores with the size dimension of 11 × 11 Å^44^, which is sufficient to accumulate S_8_ molecules (see Fig. 1a). Currently, many MOF-74(M) have been prepared, which contain Mg(II), Ca(II), Sr(II), Ba(II), Mn(II), Fe(II), Co(II), Ni(II), Cu(II), Zn(II) and Cd(II) cations as monometallic nodes^45^, or combinations thereof leading to the formation of multimetallic compounds e.g. Ni(II)/Zn(II), Mg(II)/Zn(II), Ca(II)/Zn(II), Mg(II)/Co(II) representing bimetallic compounds^46,47^, Mg(II)/Co(II)/Ni(II)/Zn(II) as tetrametallic, Mg(II)/Sr(II)/Mn(II)/Co(II)/Ni(II)/Zn(II) as hexametallic, Mg(II)/Ca(II)/Sr(II)/Mn(II)/Fe(II)/Co(II)/Ni(II)/Zn(II) as octametallic or Mg(II)/Ca(II)/Sr(II)/Ba(II)/Mn(II)/Fe(II)/Co(II)/Ni(II)/Zn(II)/Cd(II) as decametallic MOF-74^48^. The central atom is pentacoordinated by four oxygen atoms of the DOBDC^4−^ ligands, utilizing all of their oxygen atoms in coordination bonds with other metal ions, and the fifth coordination site is occupied by water molecule to form a distorted tetragonal pyramid geometry around the central atom (see Fig. 1b). The removal of coordinated water molecules results in the formation of coordinatively unsaturated sites (CUSs), representing Lewis acidic sites, which play an important role as primary adsorption or catalytic active sites. MOF-74-type compounds feature a high density of CUSs lying in the corners of 1D hexagonal channels along the *c* crystallographic axis, as depicted in Fig. 1a. CUSs or Lewis acidic sites facilitate strong binding of CO_2_, H_2_, CH_4_, S_x_ and other molecules, acting as Lewis bases, to these sites^44,45,49,50^. Furthermore, the solvothermal synthesis of MOF-74(M) is relatively straightforward and can be achieved under mild conditions, which also enables facile alteration of the central metal within the same framework. A practical limitation, however, arises from the use of DMF as a reaction solvent, since it remains strongly trapped in the pores, requires high-temperature activation for its removal, and may influence the framework geometry/stability. In the present study, this issue was resolved by a solvent-exchange process, in which DMF was replaced with methanol. Owing to its lower boiling point and weaker interaction with the framework, methanol can be removed more readily, thereby facilitating subsequent activation and preserving structural integrity. Even with this necessary post-synthetic treatment, the solvothermal approach to MOF-74 remains comparatively simple, relying mainly on mixing, heating, and cooling of solutions. In this context, the term ‘cost-effective’ refers to the use of inexpensive and widely available precursors, common solvents, and standard solvothermal conditions, rather than to a quantitative economic analysis. For completeness, other synthesis strategies have also been reported, including solvent-free mechanochemical synthesis^51^, one-pot^52^, microwave^53^, vapour-assisted^54^, and dry gel synthesis^55^.

Herein, a composite material of MOF-74, carbon black, and sulphur was designed as a long-lasting electrode material for Li–S batteries, S/MOF-74(M). MOF-74 based on Ni(II), Mg(II), and Fe(II) metal ions were synthesized using the conventional solvothermal method, and cathode material based on sulphur was prepared. MOF-74 has a large number of micropores, which is beneficial for the physical confinement and capture of sulphur. Moreover, as shown in Fig. 1a, the pore size is sufficient to store sulphur molecules. The main objective was to study the influence of different central atoms in MOF-74 on the conductivity of electrode material and the chemical confinement of sulphur. Fe(II) ions, together with Mg(II) cations, were chosen because of their low toxicity and cost, relatively high availability and abundance in nature (fourth and seventh, respectively). In addition, Mg(II) ions have the advantage of high gravimetric/specific capacity as they have the lowest atomic weight of all the selected metal ions. Although Ni(II) ions lag behind in the above properties compared to Mg(II) and Fe(II) cations, the high catalytic properties of Ni have been proven by application in several types of batteries. Ni has been widely used in batteries since the 1980s in nickel–cadmium (Ni–Cd) and nickel metal hydride (NiMH) devices^58,59^. Two of the most commonly used types of Li-ion batteries, i.e. Nickel Cobalt Aluminium (NCA) and Nickel Manganese Cobalt (NMC), use at least 33% nickel with a tendency to increase its amount^60^. On the other hand, the advantage of Fe(II) and Ni(II) ions compared to Mg(II) cations is their variability of oxidation states (II/III), which can also help to achieve stability in the kinetics of electrochemical processes. Moreover, the overall porous network may mitigate the volumetric expansion of sulphur during cycling. The prepared S/MOF-74(Ni) composite reached an extremely stable cycle performance which is discussed in detail and compared with Mg(II) and Fe(II) MOF-74 analogues and other sulphur cathodes containing MOF in Li–S batteries.

## Experimental

### Chemicals

2,5-dihydroxy-1,4-benzenedicarboxylic acid (H_4_DOBDC, 98%, Merck), manganese(II) nitrate hexahydrate (Mg(NO_3_)_2_·6H_2_O, 98%, Lachema), nickel(II) nitrate hexahydrate (Ni(NO_3_)_2_·6H_2_O, 98.5%, Merck), iron(II) chloride tetrahydrate (FeCl_2_·4H_2_O, 98%, Sigma-Aldrich), *N,N´*-dimethylformamide (DMF, ≥ 99%, Sigma-Aldrich), ethanol (96%, Centralchem), 2-propanol (99%, Merck), methanol (98%, Centralchem) were used for the synthesis and solvent-exchange process of MOF-74 materials. Sulphur (99.98%, Sigma-Aldrich), Super P (≥ 99%, Timcal), polyvinylidene fluoride (PVDF, average Mw ~ 534,000 by GPC, Sigma-Aldrich), *N*-methyl-2-pyrrolidone (NMP, 99.5%, Sigma-Aldrich), 1,2-dimethoxyethane (DME, 99.5%, Sigma-Aldrich), 1,3-dioxolane (DOL, 99.8%, Sigma-Aldrich), metal lithium (99.9%, Sigma-Aldrich), lithium nitrate (LiNO_3_, 99.99%, Sigma-Aldrich), lithium bis(trifluoromethanesulfonyl) imide (LiTFSI, 99.99%, Sigma-Aldrich) were used for electrode preparation, electrolyte and cell assembly. NMR for 2,5-dihydroxy-1,4-benzenedicarboxylic acid (H_4_DOBDC): ^**1**^**H NMR** (400 MHz, DMSO) *δ*: 7.27 (2H, s, H-3, H-6); ^**13**^**C NMR** (100 MHz, DMSO) *δ*: 117.4 (C-3,C-6), 119.4 (C-1, C-4), 152.1 (C-2, C-5), 170.4 (C=O), 170.5 (C=O) (see Fig. S1 in ESI).

### Synthesis and methanol-exchange process of MOF-74(M) (M = Mg, Fe and Ni)

MOF-74(M) (M = Mg(II), Fe(II) and Ni(II)) materials were prepared by modifying the synthetic procedures published in the literature^61,62^.

MOF-74(Mg and Ni): H_4_DOBDC (2,5-dihydroxy-1,4-benzenedicarboxylic acid, 0.111 g, 0.559 mmol for MOF-74(Mg); 0.478 g, 2.41 mmol for MOF-74(Ni)) was added in 70 cm^3^ of *N,N´*-dimethylformamide (DMF) in a glass Ace® front seal pressure tube. The suspension was stirred until the H_4_DOBDC was completely dissolved. Subsequently, nitrate of the given metal was added to the solution (Mg(NO_3_)_2_·6H_2_O, 0.475 g, 1.85 mmol for MOF-74(Mg); Ni(NO_3_)_2_·6H_2_O, 2.378 g, 8.18 mmol for MOF-74(Ni)), 70 cm^3^ of ethanol, 70 cm^3^ of water and the suspension was stirred until homogeneous. The Ace® pressure tube was capped and placed in an oven and heated at 125 °C for MOF-74(Mg) and 100 °C for MOF-74(Ni) with a heating rate of 1 °C min^−1^ for 24 h. Subsequently, the reaction mixture was cooled to ambient temperature with a cooling rate of 0.1 °C min^−1^. The obtained as-synthesized yellow (MOF-74(Mg) (AS)) and yellow–brown (MOF-74(Ni) (AS)) microcrystalline powders were filtered, washed with methanol and dried in an airflow (yield: 93% for MOF-74(Mg) and 87% for MOF-74(Ni) based on H_4_DOBDC, determined from the weight of the solvent-free material).

MOF-74(Fe): H_4_DOBDC (200 mg, 1.0 mmol) was added in 18.5 cm^3^ of DMF in a glass Ace® front seal pressure tube. The solution was ultrasonicated for 5 min until the complete dissolution of H_4_DOBDC. Subsequently, FeCl_2_·4H_2_O (400 mg, 2.0 mmol), 1 cm^3^ of 2-propanol and 1 cm^3^ of water were added to the solution, and the suspension was ultrasonicated for 15 min until the ferric chloride tetrahydrate was completely dissolved. The homogeneous reaction mixture was placed in an oven with controlled heating, while the temperature regime was set as follows: The solution was heated to a temperature of 105 °C with a heating rate of 1 °C min^−1^, while the reaction took place at the given temperature for 24 h. Subsequently, the reaction mixture was cooled to ambient temperature with a cooling rate of 0.1 °C min^−1^. The resulting as-synthesized dark purple needle-like crystals of MOF-74(Fe) (AS) were filtered, washed with methanol and dried in an air stream (yield: 88% based on H_4_DOBDC, determined from the weight of the solvent-free material).

The prepared materials were subjected to a solvent exchange process (EX) due to the removal of DMF molecules from the pores of as-synthesized (AS) materials. DMF with a high boiling point (b.p. 153 °C) was replaced by methanol (b.p. 65 °C) due to easier activation for nitrogen adsorption measurements and sulphur encapsulation in the preparation of electrode materials. As-synthesized (AS) materials were stored in methanol for 2 weeks, with the methanol being changed every 2 days. Samples after methanol exchange are designated in the text as MOF-76(Mg) (EX), MOF-76(Fe) (EX) and MOF-76(Ni) (EX).

### Electrode preparation and cell assembly

Cathode materials with sulphur were prepared using MOF-74 as a matrix for sulphur. Sulphur, activated MOF-74(M) (M = Mg(II) at 200 °C, Fe(II) at 60 °C and Ni(II) at 250 °C) and carbon Super P® were milled in a planetary ball mill using a zirconium oxide grinding jar and milling balls at 500 rpm for 30 min. To prepare the electrode slurry, polyvinylidene fluoride (PVDF) was dissolved in an *N*-methyl-2-pyrrolidone (NMP), and the mixture of sulphur, MOF-74(M) (M = Mg(II), Fe(II) and Ni(II)), and Super P® was added. The resulting electrode composition for sulphur, MOF-74(M) (M = Mg(II), Fe(II) and Ni(II)), Super P®, and PVDF was 60:15:15:10 (mass ratio). The electrode slurry was mixed on a magnetic stirrer for 24 h and uniformly dispersed on an aluminium current collector with a carbon coating, and the slurry was dried at 60 °C for 24 h. The electrodes were punched onto 18 mm diameter round disks, pressed at a pressure of 300 kg cm^−2^, and dried under vacuum for 12 h and in the oven in a glove box at 60 °C for 24 h. The sulphur mass loading was controlled at around 2.7 mg cm^−2^. The laboratory test cells El-Cell® were used for electrochemical characterization. The cells were assembled using a glass fibre separator, lithium metal anode, and electrolyte in a composition of 1,2-dimethoxyethane (DME) and 1,3-dioxolane (DOL) (2:1 volume ratio) with 0.25 M of lithium nitrate (LiNO_3_) and 0.7 M of lithium bis(trifluoromethanesulfonyl) imide (LiTFSI). The high-purity argon-filled glovebox Jacomex was used to assemble the cells.

### Lithium polysulfide adsorption test

The visual polysulfide adsorption test was conducted by adding an equivalent amount (10 mg) of MOF-74(M)/C composite to 2 mL of lithium polysulfide solution (5 mmol L^−1^). The Li_2_S_6_ solution was prepared inside a glove box by dissolving stoichiometric amounts of sulfur and lithium sulfide (Li_2_S) in a mixed solvent of DME/DOL (2:1 v/v) at 60 °C.

### Methods and characterization

Fourier-transform infrared (FTIR) spectra of the MOF-74 materials, their corresponding composite cathodes and individual componets were recorded using a Thermo Scientific AVATAR 6700 spectrometer in the wavenumber range of 3100–400 cm^−1^. The measurements were carried out in transmission mode using KBr pellets. Prior to sample preparation, spectroscopic-grade KBr was thoroughly dried at 600 °C to eliminate residual moisture. The pellets were prepared by homogenizing the sample with KBr in a 1: 100 weight ratio (sample: KBr) and pressing the mixture into transparent disks. Each spectrum was acquired by averaging 64 scans at a spectral resolution of 2 cm^−1^.

Thermogravimetric analysis (TGA) was carried out on a TGA Q500 apparatus. All measurements were performed in air with a flow rate of 40 cm^3^ min^−1^ using a platinum pan. The heating ramp was set at 10 °C min^−1^ in the temperature range of 30–600 °C.

Powder X-ray diffraction experiments (PXRD) were performed to study the phase purity and stability of MOF-74 frameworks before/after the solvent exchange process, activation and cathode materialsʼ preparation. The analysis was carried out on a D2 PHASER diffractometer from Bruker using CuKα radiation (*λ* = 1.54056 Å) in the *2θ* range from 10 to 50° with a 0.1° step at a scan speed of 0.5° min^−1^.

Nitrogen physisorption analysis was conducted to assess the porous characteristics of the solvent exchanged (EX) samples and cathode materials, following the procedure outlined in the literature^63,64^. Prior to performing the physisorption experiments, the methanol-exchanged (EX) samples were degassed at different temperatures of 60, 200 and 250 °C for 20 h under a dynamic vacuum. In the case of the composite cathode materials (S/MOF-74), degassing was carried out at 60 °C to avoid sulfur melting, as elemental sulfur melts at approximately 114 °C and higher activation temperatures could lead to loss of active material. The adsorption isotherms were recorded over a relative pressure range of approximately 10^−4^ − 0.995 *p*/*p*_0_ (relative pressure). The nitrogen adsorption isotherms were used to calculate the BET area (*S*_BET_). The total pore volume was calculated from the pore size distribution (PSD) curves obtained by fitting the experimental adsorption data with a Non-Local Density Functional Theory (NLDFT) adsorption kernel (ASiQwin software, Quantachrome Instruments).

The materials were characterized for their chemical composition (C, H, N, and S in wt.%) using a Vario MICRO CHNS analyzer (Elementar Analysensysteme GmbH, Germany). The analysis is based on the complete combustion of the sample at 1800 °C, followed by separation of the resulting gases through a temperature-controlled desorption column to ensure peak resolution without overlap.

NMR spectra were recorded on a Varian VNMRS 600 instrument operating at 599.87 MHz for ^1^H and 150.84 MHz for ^13^C. Chemical shifts (*δ*, ppm) are referenced to the residual solvent signals: DMSO-d_6_ at 39.5 ppm for ^13^C and DMSO-d_5_ at 2.5 ppm for ^1^H.

High-resolution X-ray photoelectron spectroscopy (XPS) analyses were performed using a SPECS PHOIBOS 100 spectrometer equipped with an Al Kα radiation source (200 W), under ultra-high vacuum conditions (base pressure ~ 10^−8^ mbar). The samples were mounted on a molybdenum holder using conductive carbon tape to ensure proper electrical contact.

The morphology of the samples was investigated using scanning electron microscopy (SEM) on Tescan Lyra3 and Tescan Vega3, and energy-dispersive X-ray spectroscopy (EDX) was performed using Bruker XFlash 5010 detector.

The galvanostatic cycling measurements and cyclic voltammetry (CV) were performed on a BioLogic VMP3 potentiostat. The CV was measured at a scan rate of 0.1 mV s^−1^ and in a potential window from 1.8 to 3.0 V. Galvanostatic cycling was performed with the potential range of 1.8–2.8 V (vs Li/Li^+^). The gravimetric capacity refers to the mass of sulphur in the electrode. Electrochemical impedance spectroscopy (EIS) was measured in the frequency range of 1 MHz–100 mHz with an amplitude of 10 mV and EIS spectra were fitted in MATLAB. In order to study the shuttle effect, shuttle current measurement by a direct method based on^6,65^ was performed. Two cycles at 0.1 C were performed to reset the battery history and analyse the actual capacity of the cell. The cells were fully charged up to 2.8 V rested for 30 min to allow battery to stabilize. Due to the rest, the measurement at 0% depth-of-discharge (DOD) was not possible, and we measured at 2% DOD instead. Subsequently, after the battery rest, constant voltage was applied for 2 h, and current was monitored. The current value stabilised during these 2 h and the last current value is considered as the shuttle current. The next step was discharge to 90% and measurement of the shuttle current at this DOD stage. This procedure was repeated after 100 cycles.

## Results and discussion

### Thermogravimetric analysis

The thermal stability and desolvation of all the prepared materials were evaluated by thermogravimetric analysis (TGA), both for AS materials and after the EX process (see Fig. S2 in ESI). TGA profiles showed weight losses consistent with previous findings described in the literature^66^.

In the case of as-synthesized materials, during the initial step in the temperature range of 30–100 °C, the mass change showed a rapid decrease up to 5–7 wt.% with increasing temperature. This is attributed to the release of physisorbed solvents used in the synthesis (ethanol, water and DMF) from the samples´ surface. Subsequently, in the second stage and the temperature range of 80–250 °C for MOF-74(Mg), 80–180 °C for MOF-74(Fe) and 100–220 °C for MOF-74(Ni), the decrease in mass change (approx. 20 wt.%) proceeded more gradually with continued heating, mainly due to the removal of coordinated water molecules and crystallization DMF from the pores, thereby revealing the coordinatively unsaturated sites (CUSs). In the third decomposition stage at temperatures exceeding 400 °C, 200 °C and 250 °C for MOF-74(Mg), MOF-74(Fe) and MOF-74(Ni), respectively, led to a sustained 35–40 wt.% change, indicating a structural collapse due to the decomposition of DOBDC^4−^ linker.

In the case of solvent exchange materials, MOF-74(M) (EX) (M = Mg(II), Fe(II), Ni(II)), a mass decrease can be observed at the beginning of thermal decomposition in the temperature interval of 30–250 °C, corresponding to the release of methanol from the channel system and coordinated water molecules. Above this temperature, some compounds exhibit a plateau, and further heating causes the collapse of frameworks. MOF-74(Mg) exhibited the highest thermal stability among the prepared compounds (see Fig. S2a in ESI). In the temperature interval of 30–250 °C, solvent removal (35 wt.%) from the compound occurs. Subsequently, the desolvated framework is stable in the 250–400 °C interval, and above 400 °C, thermal decomposition of the polymer skeleton is observed. MOF-74(Fe) exhibited the lowest thermal stability among the three prepared materials. A continuous decrease in mass change (20 wt.%) from 30 to 200 °C can be observed on the TGA curve (see Fig. S2b in ESI). Above this temperature, decomposition and collapse of the framework occurs. On the TGA curve of MOF-74(Ni) (EX) (see Fig. S2c in ESI), a rapid mass loss (35 wt.%) was observed from 30 to 220 °C with the presence of a plateau up to 300 °C, and above this temperature, the framework collapsed.

Based on the above TGA results for solvent exchanged samples, the thermal stability of the prepared materials can be arranged in the following order: MOF-74(Mg) (EX) − 400 °C ˃ MOF-74(Ni) EX − 300 °C ˃ MOF-74(Fe) (EX) − 200 °C. The above information regarding the framework stability was crucial to determining the activation temperature of the materials for subsequent nitrogen adsorption/desorption measurements and calculation of textural properties.

### Nitrogen adsorption/desorption measurements

**Fig. 2:**
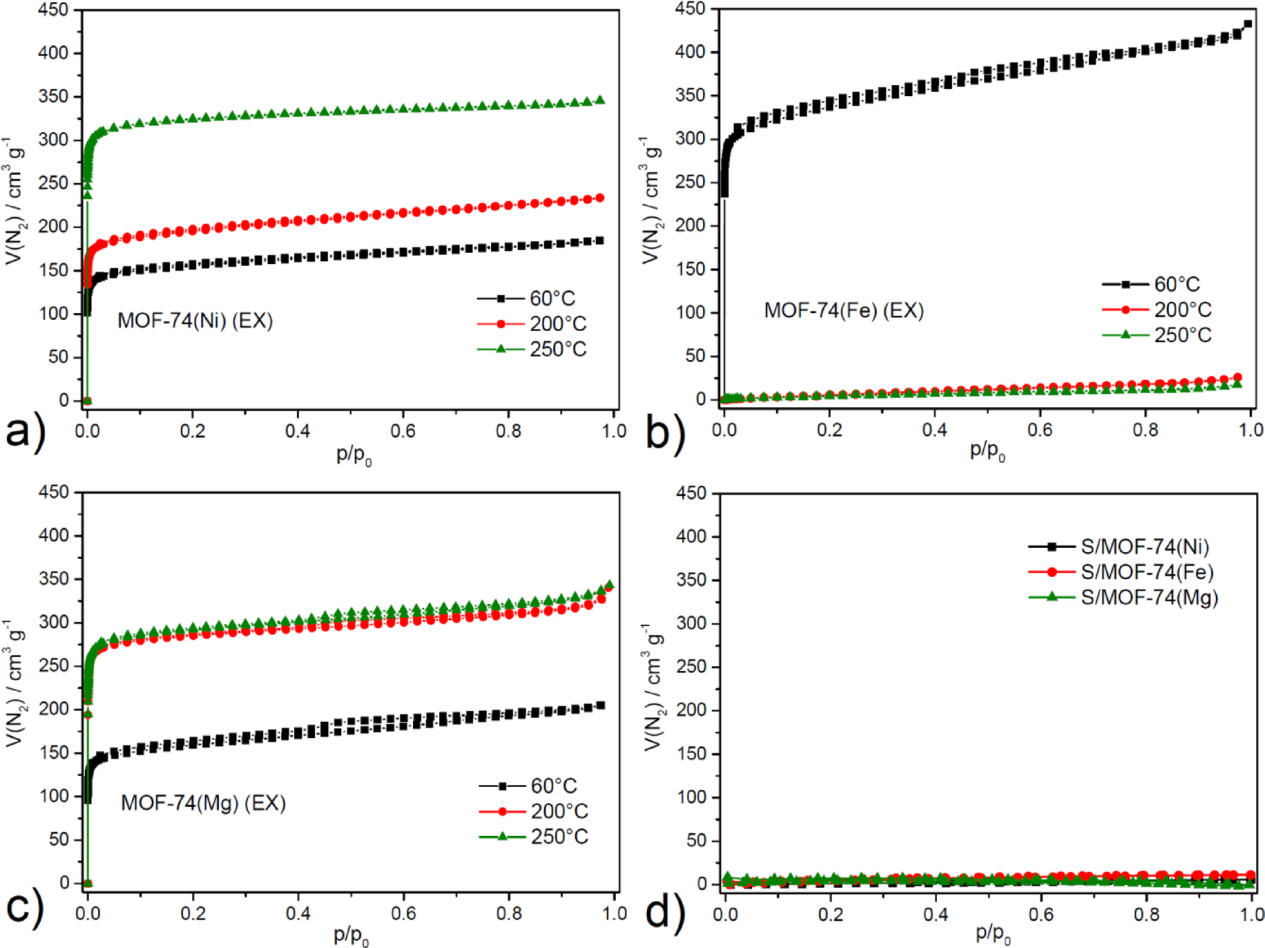
Nitrogen adsorption/desorption isotherms measured at − 196 °C for methanol-exchanged (**a**) MOF-74(Mg), (**b**) MOF-74(Fe), (**c**) MOF-74(Ni) samples activated at 60, 200, 250 °C and (**d**) electrode materials S/MOF-74(M) (M = Mg(II), Fe(II) and Ni(II)).

The specific surface area of the sulfur host plays a crucial role in determining sulfur distribution and electrolyte accessibility. A larger surface area facilitates more effective sulfur confinement, enhances sulfur–electrode contact, and improves electrochemical kinetics. Additionally, it provides more active sites for the adsorption of lithium polysulfides, thereby helping to suppress the shuttle effect. However, an excessively high surface area, in the absence of adequate electrical conductivity or chemical anchoring sites, may promote undesirable side reactions and accelerate electrolyte decomposition. Consequently, achieving an optimal balance between surface area, conductivity, and chemical functionality is essential for maximizing battery performance. To comprehend the textural properties (specific surface area (*S*_BET_), pore volume (*V*_p_) and size diameter (*d*)) of the material in order to find the ideal activation temperature, adsorption/desorption measurements were conducted with nitrogen as a probe molecule at − 196 °C. The obtained adsorption/desorption isotherms are depicted in Fig. 2a–c, and the calculated values for *S*_BET_, *V*_p_, and *d* are summarized in Table 1. MOF-74(M) (M = Mg(II), Fe(II) and Ni(II)) exhibited a typical type* I* isotherm in accordance with the IUPAC technical report^67^, indicating the presence of narrow micropores smaller than 1.0 nm. This suggests enhanced adsorbent-adsorptive interactions within the frameworks containing micropores. Additionally, type *H3* hysteresis loops were observed in the relative pressure range of 0.4 to 0.7, indicating that all solvent-exchanged materials lie on the interface between microporous and mesoporous structures, potentially arising from crystal defects, which do not degrade the sample quality.

**Table 1:** Summary of the calculated textural properties (specific surface area (*S*_BET_), pore volume (*V*_p_) and pore diameter (*d*)) for MOF-74(M) (M = Mg(II), Fe(II) and Ni(II)) samples activated at 60, 200 and 250 °C and composite cathode materials (S/MOF-74(M)) activated at 60 °C based on of the results of nitrogen adsorption/desorption measurements.

	Activation temperature (°C)	*S*_BET_ (m^2^ g^−1^)	*V*_p_ (cm^3^ g^−1^)	*d* (nm)
MOF-74(Mg) (EX)	60	616	0.287	0.989
	200	1154	0.467	0.984
	250	1175	0.453	0.984
S/MOF-74(Mg)	60	17	0.033	3.775
MOF-74(Fe) (EX)	60	1307	0.594	1.038
	200	9	0.036	2.769
	250	7	0.024	2.647
S/MOF-74(Fe)	60	5	0.002	3.822
MOF-74(Ni) (EX)	60	612	0.259	1.41
	200	768	0.327	0.984
	250	1315	0.480	0.984
S/MOF-74(Ni)	60	9	0.029	3.179

Based on the adsorption measurements of methanol-exchanged materials MOF-74(M) (M = Mg(II), Fe(II) and Ni(II)), it was observed that increasing the activation temperature significantly influences the *S*_BET_ values. For MOF-74(Mg) (EX) and MOF-74(Ni) (EX), the samples exhibit similar behaviour, whereby an increase in activation temperature leads to an increase in specific surface area, from 616 m^2^ g^−1^ at 60 °C to 1175 m^2^ g^−1^ at 250 °C for MOF-74(Mg) (EX) and 612 m^2^ g^−1^ at 60 °C to 1315 m^2^ g^−1^ at 250 °C for MOF-74(Ni) (EX). In contrast, the MOF-74(Fe) (EX) sample shows an inverse relationship with activation temperature, as higher temperatures result in the adsorption of a limited amount of gas. The maximum specific surface area was achieved at an activation temperature of 60 °C with an *S*_BET_ of 1307 m^2^ g^−1^ for MOF-74(Fe) (EX). These findings are consistent with the results of the thermal analysis described below. As can be seen from Table 1, the pore volume of the materials increased with increasing specific surface area. Positive information is that the calculated pore diameter from the adsorption measurements (9.84 Å for MOF-74(Mg), 10.38 Å for MOF-74(Fe) and 9.84 Å for MOF-74(Ni)) is close to the theoretical value of 11 Å determined from the crystal structure. The requirements for the sulphur host are a high surface area due to more available internal surface area, leading to better sulphur distribution and confinement. Therefore, the activation temperatures with the highest surface areas of MOF materials were used prior to sulphur-based cathode preparation. These conditions provide the most favorable internal surface area for sulfur infiltration, which is essential for achieving high utilization and confinement efficiency in Li–S cathodes.

All observed *S*_BET_ and other textural properties are in good agreement with many publications, which has also been confirmed in the study of bimetallic MM´-MOF-74 (where MM´ is a combination of two cations selected from a set of Co(II), Ni(II), Cu(II), Ca(II), Zn(II), Mg(II) ions)^68^.

In addition to the pristine MOF-74 materials, nitrogen adsorption/desorption measurements were also performed for the S/MOF-74(M) (M = Mg(II), Fe(II) and Ni(II)) composite cathodes to assess the changes in textural properties upon sulfur incorporation. The results presented in Fig. 2d and Table 1 reveal a substantial decrease in specific surface area, pore volume, and a notable shift in apparent pore size when compared to the activated MOF-74 materials.

For all three systems (S/MOF-74(Mg), S/MOF-74(Fe), and S/MOF-74(Ni)) the *S*_*BET*_ values dropped dramatically, indicating that sulfur occupies a significant portion of the accessible pore space. Specifically, *S*_*BET*_ values decreased from 1175 to 17 m^2^ g^−1^ for Mg(II)-based, from 1307 to 5 m^2^ g^−1^ for Fe(II)-based, and from 1315 to 9 m^2^ g^−1^ for Ni(II)-based composites. Correspondingly, the pore volumes declined from 0.453–0.594 cm^3^ g^−1^ in activated MOFs to as low as 0.002–0.033 cm^3^ g^−1^ in the S/MOF-74(M) (M = Mg(II), Fe(II) and Ni(II)) materials. Interestingly, the apparent pore diameter in the composites increased significantly compared to the pristine MOFs. For example, while the activated MOF-74(Ni) exhibited an average pore diameter of 0.984 nm, the S/MOF-74(Ni) composite showed an apparent diameter of 3.179 nm. Similar behavior was observed for the Fe(II)- and Mg(II)-based MOF-74 samples. This phenomenon is likely due to the altered adsorption behavior caused by partial pore blockage, reduced accessibility, and the presence of macroporous interstitial voids formed between sulfur particles and the MOF matrix^69^. These apparent larger pore sizes are therefore not representative of true structural pores but rather reflect the changed adsorption mechanism after sulfur infiltration.

The drastic reduction in surface area and pore volume, coupled with the apparent increase in pore size, collectively confirm the successful incorporation of sulfur into the MOF-74 pores. These results support the role of MOF-74 as an effective sulfur host, enabling physical confinement and distribution of sulfur within the microporous framework, which is essential for suppressing the shuttle effect and enhancing the performance of Li–S battery cathodes.

### Elemental analysis

Elemental analysis was performed to determine the chemical composition of a series of MOF-74 materials differing in their metal centers (Fe(II), Ni(II), Mg(II)) and post-synthetic treatment (as-synthesized (AS) vs. solvent-exchanged (EX)). The samples were further subjected to thermal activation at varying temperatures (60 °C, 200 °C, and 250 °C), and the effect of temperature on the elemental composition was evaluated. The results are summarized in Table S1 in ESI, which includes both the proposed empirical formulas and those derived from experimental elemental compositions (%C, %H, %N), alongside theoretical values calculated for idealized compositions.

For the as-synthesized and methanol-exchanged materials without thermal activation, the measured elemental compositions show good agreement with the calculated values. This confirms the expected incorporation of the organic linker (2,5-dihydroxyterephthalate), metal ions (Fe(II), Ni(II), Mg(II)), and the presence of guest molecules (DMF and water for AS; MeOH for EX samples) in the pores. However, upon thermal activation, discrepancies between the calculated and experimental values begin to emerge, reflecting the progressive removal of guest molecules (MOF-74(Ni) (EX) and MOF-74(Mg) (EX)) and, in some cases, partial decomposition of the framework (MOF-74(Fe) (EX)). These trends correlate well with thermogravimetric analysis (TGA) and nitrogen physisorption data, which independently establish the optimal activation temperatures for each material (60 °C for MOF-74(Fe), 250 °C for MOF-74(Ni) and 200 or 250 °C for MOF-74(Mg)).

The Fe(II)-based MOF-74 (EX) shows the best match between calculated and experimental elemental compositions when activated at 60 °C, indicating the successful removal of pore solvents while preserving the composition of the framework. At higher temperatures (200 °C and 250 °C), a significant drop in carbon and hydrogen content is observed, suggesting the onset of framework degradation.

For the sample MOF-74(Ni) (EX), a gradual decrease in carbon and hydrogen content is observed with increasing activation temperature (60 °C → 200 °C → 250 °C), corresponding to the stepwise loss of methanol from the pores. Only at 250 °C is the experimentally determined composition in close agreement with the calculated formula for the fully activated material. This confirms that complete solvent removal is achieved at this temperature, which is consistent with both TGA and sorption analysis.

MOF-74(Mg) (EX) reaches the expected stoichiometry upon activation at 200 °C, with no significant changes in elemental composition upon further heating to 250 °C. This suggests that the solvent removal process is completed at 200 °C, and the framework remains thermally stable up to at least 250 °C. This behavior also aligns well with the corresponding TGA and nitrogen adsorption results.

The combined data reveal that the optimal activation temperature is highly metal-dependent. The Fe(II)-based MOF requires only mild activation (60 °C) to remove solvent molecules without degrading the structure, while Ni(II)- and Mg(II)-based MOF-74 materials tolerate higher activation temperatures (250 °C and 200–250 °C, respectively). Notably, the inability to assign clear molecular formulas in certain samples reflects the gradual and often non-stoichiometric release of guest molecules during activation, which is a common challenge in the interpretation of elemental analysis for porous materials.

Elemental analysis was also performed to verify the sulfur content in the composite electrode materials S/MOF-74(Mg), S/MOF-74(Fe), and S/MOF-74(Ni), where sulfur was incorporated into the corresponding electrode materials. The theoretical sulfur content in these composites was estimated to be around 60 wt.%, which represents the targeted loading necessary for optimal electrochemical performance. As summarized in Table S2 in ESI, the experimentally determined sulfur contents are in close agreement with the expected value: S/MOF-74(Mg): 58.64%; S/MOF-74(Fe): 60.88%; S/MOF-74(Ni): 59.57%. These results confirm the successful incorporation of sulfur into the S/MOF-74-based electrode materials. The slight deviations from the theoretical value are within acceptable analytical margins.

### Infrared spectroscopy

**Fig. 3:**
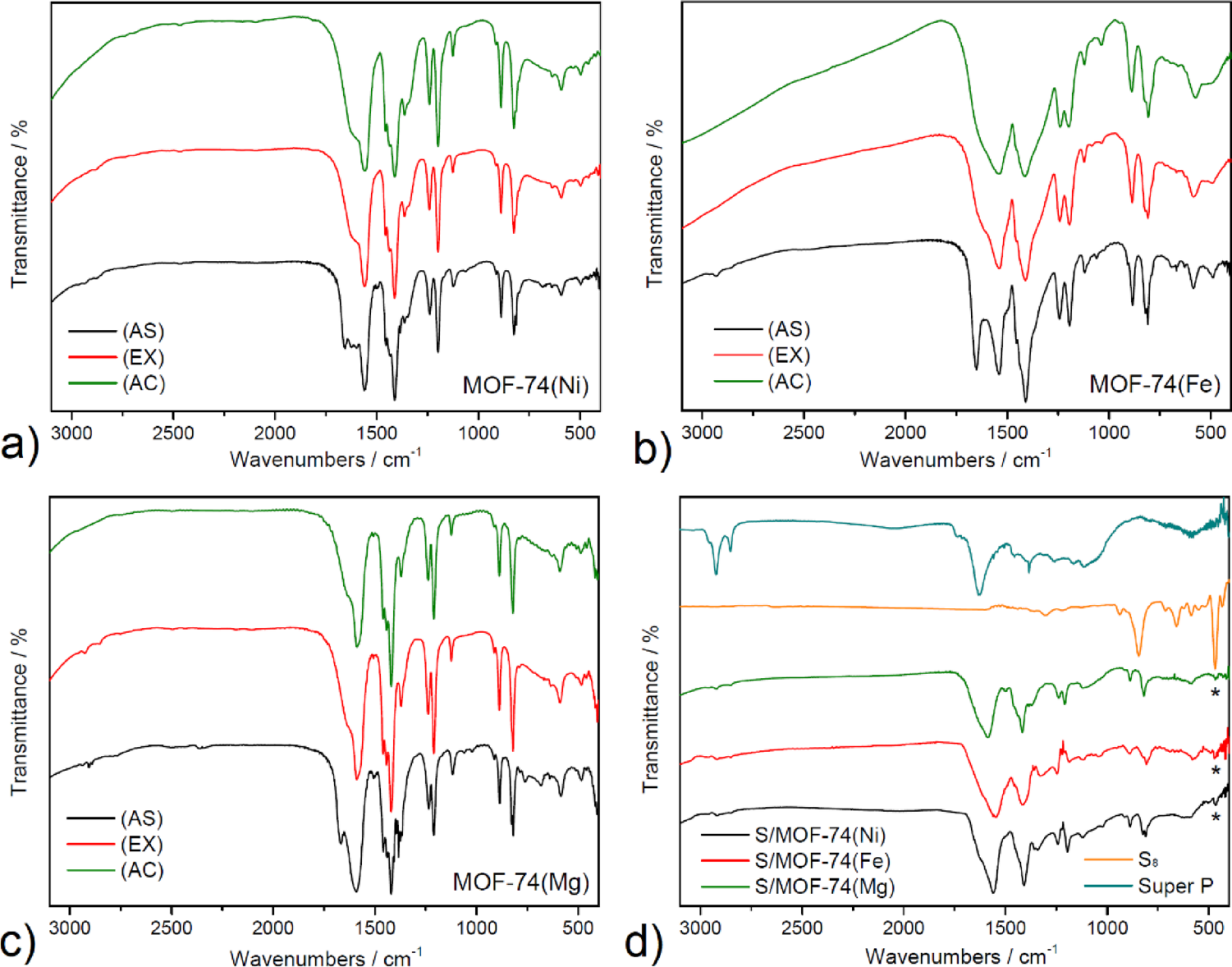
Infrared spectra of AS, EX and AC MOF-74(M), where M = (**a**) Ni(II), (**b**) Fe(II) and (**c**) Mg(II) and (**d**) cathode materials S/MOF-74(M) materials (* refers to *δ*(SSSS) vibration) with S_8_ and Super P.

FTIR spectroscopy was used to examine the chemical structure and confirm the presence of functional groups in the MOF-74(M) (M = Mg(II), Fe(II) and Ni(II)) materials in their as-synthesized (AS), methanol-exchanged (EX), and activated (AC) forms, as well as in the sulfur-based composite cathodes S/MOF-74(M). The recorded spectra are presented in Fig. 3a–d with vibrational band assignments summarized in Table 2.

**Table 2:** Assignment of major characteristic absorption bands to wavenumbers (in cm^−1^) for MOF-74(M) (AS), (EX), (AC) and cathode materials S/MOF-74(M) (M = Mg(II), Fe(II) and Ni(II)).

	*ν*(CH)_aliph_	*ν*(C = O)	*ν*_*as*_(COO^−^)	*ν*(CC)_ar_	*ν*_*s*_(COO^−^)	*δ*(COO^−^)	*δ*(SSSS)
MOF-74(Ni) (AS)	2937 2889	1657	1559	1500	1410	823	–
MOF-74(Ni) (EX)	2858	–	1561	–	1410	825	–
MOF-74(Ni) (AC)	–	–	1559	–	1410	826	–
S/MOF-74(Ni)	2923 2855	–	1560	–	1408	823	469
MOF-74(Fe) (AS)	2936 2890	1654	1542	–	1410	809	–
MOF-74(Fe) (EX)	2854	–	1542	–	1411	807	–
MOF-74(Fe) (AC)	–	–	1542	–	1412	809	–
S/MOF-74(Fe)		–	1545	–	1414	807	471
MOF-74(Mg) (AS)	2940 2906 2892	1670	1589	1505	1420	819	–
MOF-74(Mg) (EX)	2926 2852	–	1589	1508	1420	820	–
MOF-74(Mg) (AC)	–	–	1590	1508	1420	819	–
S/MOF-74(Mg)	2924 2853	–	1588	1503	1418	820	470
S_8_	–	–	–	–	–	–	471
Super P	2958 2924 2853	–	–	1560 1540 1506	–	–	–

ar, Aromatic; aliph, Aplihatic; s, Symmetric; as, Asymmetric.

Across all samples containing MOF-74, characteristic absorption bands corresponding to the 2,5-dihydroxyterephthalate linker (DOBDC4^−^) were observed. These include asymmetric stretching vibrations of the carboxylate group (*ν*_*as*_(COO^−^)) appearing in the region of 1542–1590 cm^−1^, symmetric carboxylate stretching (*ν*_*s*_(COO^−^)) around 1408–1420 cm^−1^, and the deformation vibration of the carboxylate group (*δ*(COO^−^)) near 807–826 cm^−1^. In several samples, additional signals corresponding to aromatic C=C stretching vibrations (*ν*(CC)_ar_) were identified between 1500 and 1508 cm^−1^, further confirming the integrity of the aromatic linker.

In the as-synthesized (AS) materials, notable absorption bands appeared at approximately 2936–2940 cm^−1^ and 2889–2906 cm^−1^, attributed to aliphatic C–H stretching vibrations (*ν*(CH)_aliph_) arising from *N,N´*-dimethylformamide (DMF) molecules confined within the MOF pores. A distinct band in the range of 1654–1670 cm^−1^ was also observed, corresponding to the C=O stretching vibration (*ν*(C=O)) of DMF.

Upon methanol exchange (EX), the disappearance of the *ν*(C=O) band indicated effective replacement of DMF by methanol. Although aliphatic C–H stretching bands (*ν*(CH)_aliph_, 2852–2926 cm^−1^) remained in the spectra, their presence is now attributed to methyl groups of methanol. These changes confirm the successful solvent exchange process.

The FTIR spectra of the activated materials (AC) no longer exhibited any bands associated with guest solvent molecules, such as *ν*(CH)_aliph_ or *ν*(C=O), indicating their complete removal during thermal activation. Only the bands associated with the framework (COO^−^ and aromatic C=C vibrations) remained, confirming the structural preservation of the MOF framework after activation.

Composite cathode materials based on sulfur and MOF-74 (S/MOF-74(M) (M = Mg(II), Fe(II) and Ni(II))) again showed absorption bands in the aliphatic C–H region (2853–2924 cm^−1^), which were attributed to Super P conductive carbon. This assignment was confirmed by comparing the spectra with that of pure Super P, which shows similar features (see Fig. 3d). Importantly, all S/MOF-74 materials retained the key vibrational modes of the carboxylate group, specifically *ν*_*as*_(COO^−^), *ν*_*s*_(COO^−^), and *δ*(COO^−^), confirming that the MOF framework remains structurally intact following sulfur incorporation and electrode fabrication. In addition, a new weak absorption band emerged at 469–471 cm^−1^ in the S/MOF-74 composites, corresponding to the deformation vibration *δ*(SSSS) of elemental sulfur (see * in Fig. 3d)^70,71^. This signal, also observed in the spectrum of pure S₈, confirms the presence of sulfur in the composite materials. The described findings clearly confirm the successful preparation and solvent exchange process of the materials, and the assigned absorption bands are in good agreement with the published literature^72^.

Taken together, the FTIR analysis provides strong evidence for the presence and stability of the MOF-74 framework throughout synthesis, solvent exchange, and activation. It also confirms the successful removal of solvent molecules, as well as the incorporation of sulfur and conductive carbon into the final composite cathodes.

### Powder X-ray diffraction analysis

**Fig. 4:**
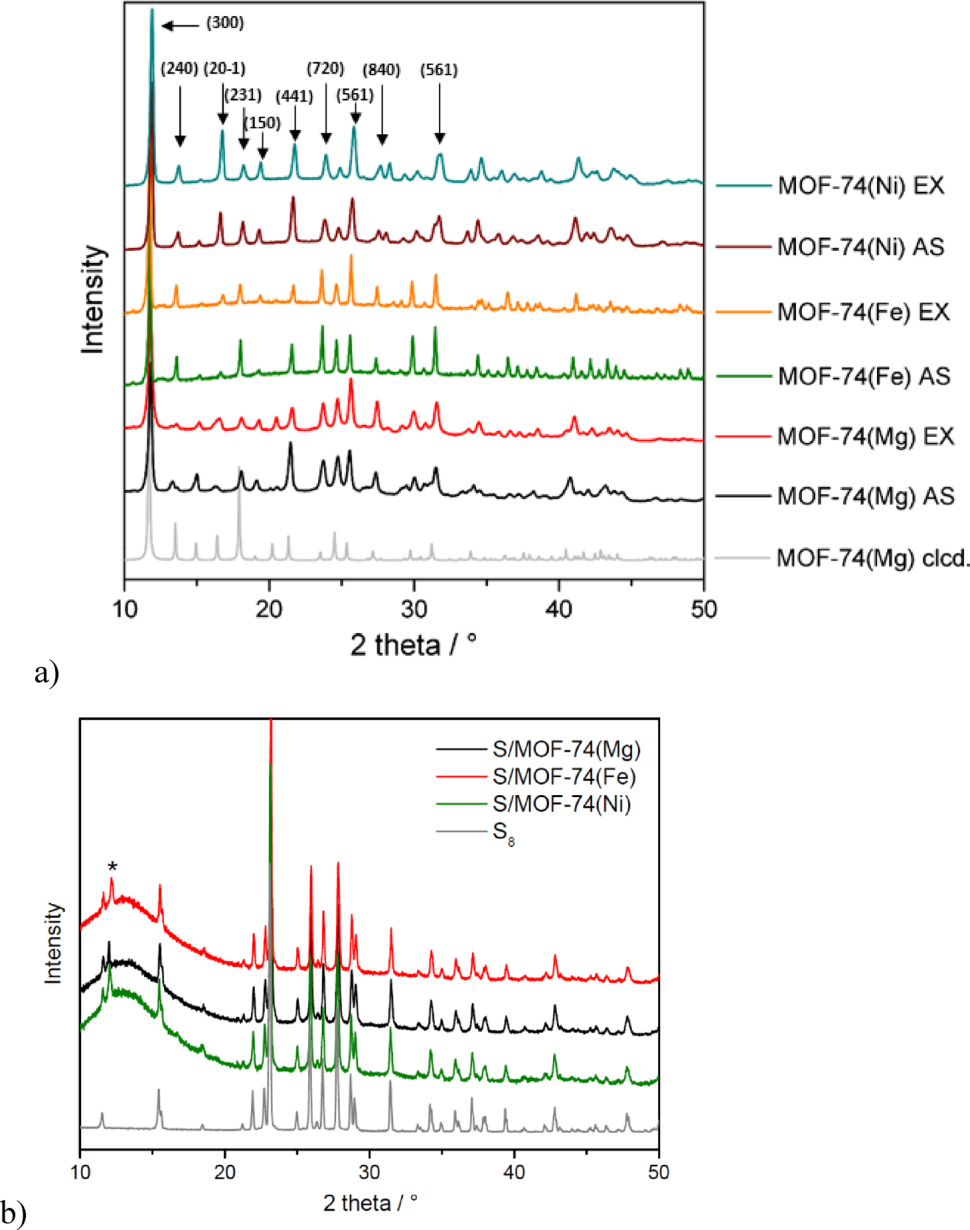
(**a**) PXRD patterns of as-synthesized (AS) and methanol-exchanged (EX) MOF-74(M) (M = Mg(II), Fe(II) and Ni(II)) samples with the designation of the corresponding crystallographic planes. (**b**) PXRD records of S/MOF-74(M) cathode materials and sulphur (the characteristic (300) reflection of MOF-74 is marked with *).

The crystal structure of as-synthetized and solvent-exchanged samples of MOF-74(M) (M = Mg(II), Fe(II) and Ni(II)) was studied using PXRD analysis, and the obtained results are shown in Fig. 4a. The measured PXRD patterns of MOF-74(M) are consistent with the calculated pattern for MOF-74(Mg), simulated from the single crystal X-ray diffraction data (see the grey curve in Fig. 4a). The compounds crystallize in the trigonal crystal system with space group R*-3* (No. 148), in agreement with previously reported structures of MOF-74 materials^73–75^. The materials revealed major diffraction peaks of *2θ* at 11.6, 14.8, 16.4, 17.9, 21.1, 24.8, 25.4, 27.4 and 31.3°, corresponding to (300), (20–1), (231), (150), (441), (720), (561), (840) and (861) crystallographic planes, respectively, which correlate well with those of reported for MOF-74(Mg)^73–75^. By comparing the diffraction patterns for EX and AS materials, it can be concluded that the solvent exchange in the MOF-74(M) (AS) porous system does not affect the crystal structure of MOF-74(M) (EX) since the PXRD records are identical for the pairs of materials.

In addition to the pristine and exchanged MOF-74 materials, PXRD patterns were also recorded for the composite cathode materials S/MOF-74(M) (M = Mg(II), Fe(II) and Ni(II)) to confirm the presence of sulfur in the prepared electrodes (see Fig. 4b). Since the electrode formulation contains a high proportion of sulfur (~ 60 wt.%), its crystalline phase dominates the diffraction pattern. The observed diffraction peaks are in agreement with those of elemental sulfur, confirming its retention in the composite structure after electrode preparation. Due to the lower weight fraction of the MOF-74 component (~ 15 wt.%) in the composite, only the most intense diffraction peak of the framework, corresponding to the (300) crystallographic plane at 2 theta angle of ~ 11.6° is visible in the PXRD pattern. This reflection, characteristic of the MOF-74 structure, is marked with * in Fig. 4b. The remaining MOF-74 reflections are not detectable, likely due to the dilution of the phase and the presence of the dominant sulfur signal. The other components of the electrode, namely Super P carbon (15 wt.%) and PVDF binder (10 wt.%), are amorphous in nature and therefore do not exhibit any distinct diffraction peaks in the PXRD patterns. The overall diffraction profiles of the composite cathodes thus reflect the crystalline sulfur phase and the presence of the MOF-74 framework, confirming the structural integrity and composition of the final electrode materials.

### X-ray photoelectron spectroscopy analysis

To investigate the chemical environment of sulfur in the S/MOF-74(M) (M = Mg(II), Fe(II) and Ni(II)) composite cathodes and to assess potential interactions between sulfur and the metal centers of the MOF frameworks, high-resolution X-ray photoelectron spectroscopy (XPS) measurements were performed. Survey spectra (see Fig. S3 in ESI) were recorded for elemental sulfur (S₈), as well as S/MOF-74(Fe), S/MOF-74(Mg), and S/MOF-74(Ni) composites, revealing the presence of characteristic peaks for sulfur, oxygen, carbon, and the respective metal centers (Fe(II), Mg(II), and Ni(II)). High-resolution spectra of the S 2*p* region were collected for all samples to determine the bonding environment of sulfur. Additionally, the Fe 2*p*, Ni 2*p*, and Mg 2*p* regions were examined for the as-synthesized and sulfur-loaded MOF-74 samples to evaluate changes in the electronic environment of the metal centers upon sulfur incorporation.

**Fig. 5:**
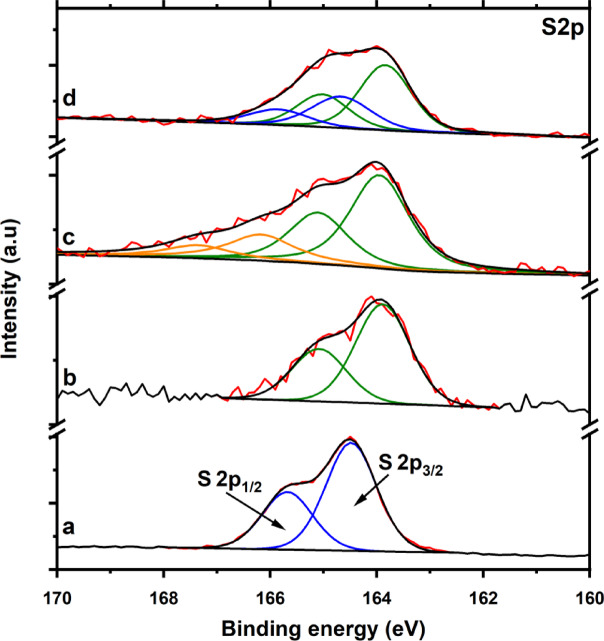
The high-resolution S 2*p* XPS spectrum of (**a**) octasulfur, (**b**) S/MOF-74(Fe), (**c**) S/MOF-74(Mg) and (**d**) S/MOF-74(Ni).

The measured S 2*p* XPS spectra of octasulfur, S/MOF-74(Fe), S/MOF-74(Mg) and S/MOF-74(Ni) are shown in Fig. 5. Figure 5a displays the S 2*p* XPS spectrum for octasulfur (S_8_), which was fitted with two peaks, S 2*p*_3/2_ at 164.48 eV and S 2*p*_1/2_ at 165.66 eV. These binding energies were assigned to sulfur in the zero oxidation state (S^0^) and serve as a reference for undisturbed sulfur environments. In comparison, the S 2*p* spectrum of the S/MOF-74(Fe) electrode material (see Fig. 5b), exhibits a similar spectral profile. The main peak was likewise fitted with two components: S 2*p*_3/2_ at 163.89 eV and S 2*p*_1/2_ at 165.07 eV. The slight shift to lower binding energies relative to pure S_8_ suggests a subtle electronic perturbation of sulfur, most likely arising from interactions between sulfur and the Fe(II) centers within the MOF framework. It is important to note that this is not a direct bond, where the shift in binding energy would be more pronounced^76–80^. A different spectral pattern was observed for the S/MOF-74(Mg) sample (see Fig. 5c), where the S2*p* envelope was deconvoluted into two sets of doublets. The first doublet with S2*p*_3/2_ at 163.95 eV and S2*p*_1/2_ at 165.10 eV is attributed to the interaction between sulfur and Mg(II) ions. The second doublet, centered at significantly higher binding energies (S2*p*_3/2_ at 166.17 eV and S2*p*_1/2_ at 167.35 eV), probably originates from partially oxidized sulfur species^81^. The S 2*p* spectrum of the S/MOF-74(Ni) material (see Fig. 5d), also displays two chemically distinct sulfur environments. The first doublet, located at 163.82 eV (S 2*p*_3/2_) and 165.00 eV (S 2*p*_1/2_), is again attributed to an interaction between sulfur and the Ni(II) centers. The second doublet, observed at 164.67 eV and 165.85 eV, closely matches the binding energies of unbound S₈, suggesting that a portion of the sulfur remains in its native form without significant electronic interaction with the MOF framework.

To gain further insight into the nature and strength of interactions between sulfur and the metal centers in the MOF-74(M) (M = Ni(II), Mg(II), Fe(II)) frameworks, the binding energy shifts in the S 2*p* doublet were analyzed. Specifically, the differences in binding energy (ǀΔǀ) between the S 2*p*_3/2_ and S 2*p*_1/2_ components of S₈ and those observed in the S/MOF-74(M) composites were evaluated. Since the S 2*p* signal consists of a spin–orbit doublet with a fixed energy separation, the ǀΔ(S 2*p*_3/2_)ǀ and ǀΔ(S 2*p*_1/2_)ǀ values are equivalent and directly reflect the magnitude of interaction between sulfur and the host framework. The calculated energy shifts (ǀΔǀ) are as follows: 0.66, 0.64 and 0.59 eV for S/MOF-74(Ni), S/MOF-74(Mg) and S/MOF-74(Fe), respectively. These results indicate that sulfur exhibits the strongest interaction with the Ni(II)-based framework, followed by moderate interaction with Mg(II) and the weakest interaction with Fe(II). The more pronounced shift observed for S/MOF-74(Ni) suggests that sulfur molecules are not merely physically confined but are also electronically stabilized within the Ni(II)-based framework, possibly via weak coordination or dipole-induced interactions. In contrast, the smaller shift in S/MOF-74(Fe) indicates limited electronic perturbation, consistent with weaker sulfur–iron(II) interactions. Importantly, this hierarchy of interaction strength is also reflected in the electrochemical performance of the composite cathodes. The enhanced sulfur–framework interaction in S/MOF-74(Ni) likely contributes to more effective sulfur immobilization and improved cycling stability, as further discussed in the electrochemical section of the manuscript.

**Fig. 6:**
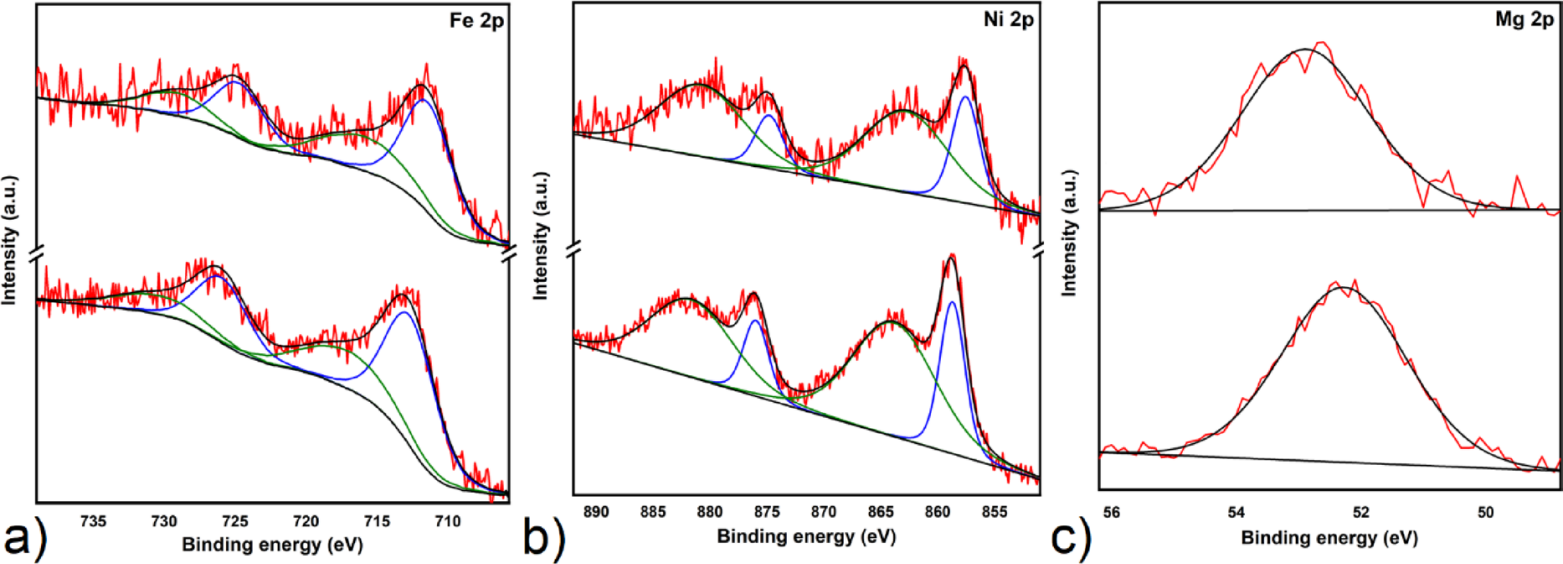
(**a**) The high-resolution XPS spectrum of (**a**) Fe 2*p* for MOF-74(Fe) (AS) (down) and S/MOF-74(Fe) (up), (**b**) Ni 2*p* for MOF-74(Ni) (AS) (down) and SMOF-74(Ni) (up) and (**c**) Mg 2*p* for MOF-74(Mg) (AS) (down) and S/MOF-74(Mg) (up).

Figure 6 presents the Fe 2*p* XPS spectra for MOF-74(Fe) samples in two different states: as-synthesized (AS) and after sulphur loading (S/MOF-74(Fe)). As shown in Fig. 6a (down), the as-prepared MOF-74(Fe) sample exhibits a Fe 2*p*_3/2_ peak at 712.62 eV and a Fe 2*p*_1/2_ peak at 725.92 eV. These peaks were assigned to iron in the (+ II) oxidation state. Additionally, satellite features are observed at 717.10 eV and 730.40 eV. Figure 6a (up) displays the Fe 2*p* spectrum for the S/MOF-74(Fe) sample, in which DMF was replaced by sulfur within the MOF pores. The spectrum maintains a similar overall shape, including both spin–orbit components and satellite peaks, but the binding energies are notably shifted to lower values. The Fe 2*p*_3/2_ and Fe 2*p*_1/2_ peaks are now located at 711.39 eV and 724.69 eV, respectively, with corresponding satellite features at 715.86 eV and 729.16 eV. This shift toward lower binding energy suggests a change in the electronic environment surrounding the Fe(II) centers. It can be attributed to the removal of strongly coordinating DMF molecules and weak electronic interactions with nearby sulfur atoms^82–84^. The removal of DMF, which coordinates to iron through more electronegative oxygen atoms, and its replacement with non-coordinating sulfur is likely to cause a shift in the Fe 2*p* XPS spectrum towards lower binding energies. This is because the electron density around the iron(II) center should increase when the more electronegative DMF ligands are no longer present.

Figure 6b compares the Ni 2*p* XPS spectra of MOF-74(Ni) (AS) and S/MOF-74(Ni). As shown in Fig. 6b (down), the as-prepared sample exhibits a Ni 2*p*_3/2_ peak at 858.71 eV and a Ni 2*p*_1/2_ peak at 875.98 eV, along with satellite features at 863.84 eV and 881.67 eV, assigned to Ni(II). The S/MOF-74(Ni) material (see Fig. 6b (up)) shows a similar spectral profile, but with all components shifted to lower binding energies. The main peaks appear at 857.54 eV (Ni 2*p*_3/2_) and 874.81 eV (Ni 2*p*_1/2_), with satellites at 862.79 eV and 880.78 eV. As with the Fe(II)-based material, this shift is attributed to the removal of DMF and subsequent weaker interaction with encapsulated sulfur^85,86^.

Figure 6c presents the Mg 2*p* XPS spectra for MOF-74(Mg) in both the as-synthesized (AS) and sulphur-loaded (S/MOF-74(Mg)) forms. As shown in Fig. 6c (down), the AS sample exhibits a Mg 2*p* peak at 52.27 eV. In contrast to the Fe(II) and Ni(II) analogues, the EX sample (Fig. 6c (up)) displays a shift to higher binding energy, with the Mg 2*p* peak located at 52.90 eV^87^.

Altogether, the XPS results provide clear spectroscopic evidence for differences in the nature and strength of sulfur–metal interactions across the MOF-74 series, which are consistent with the observed electrochemical performance trends.

### Electrochemical characterization

**Fig. 7:**
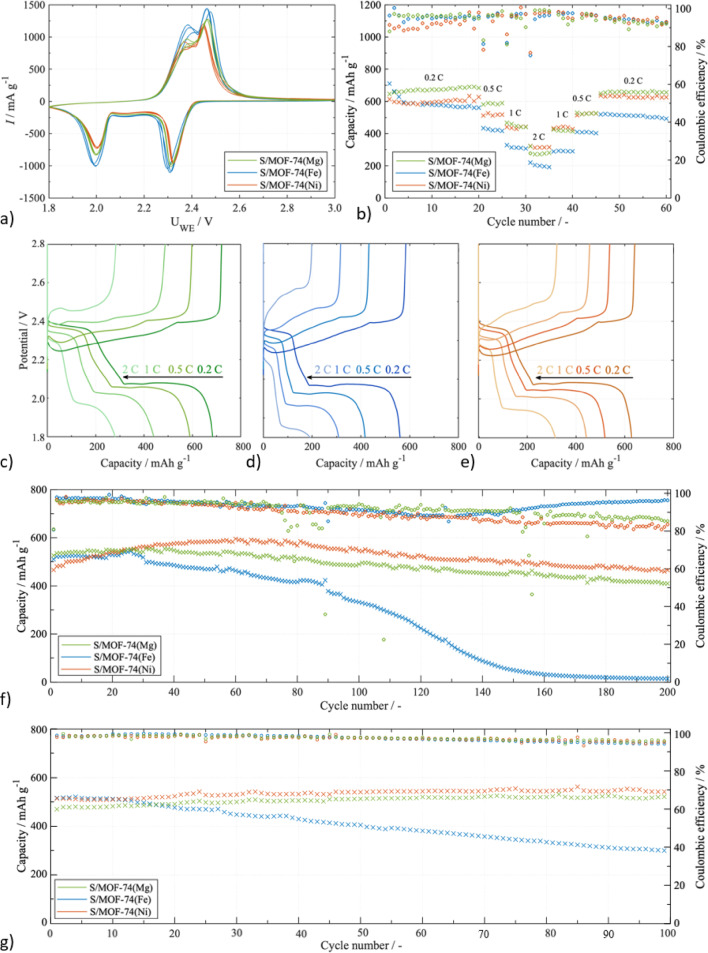
(**a**) CV curves at 0.1 mV s^−1^ of S/MOF-74(Mg), S/MOF-74(Fe), and S/MOF-74(Ni). (**b**) Rate capabilities tested at different C-rates of S/MOF-74(Mg), S/MOF-74(Fe), and S/MOF-74(Ni). Voltage profiles at various C-rates for (**c**) S/MOF-74(Mg) cathode, (**d**) S/MOF-74(Fe) cathode, and (**e**) S/MOF-74(Ni) cathode. Cycling properties and Coulombic efficiency at (**f**) 0.5 C for 200 cycles and (**g**) 1 C for 100 cycles.

To verify the electrochemical performance of MOF-74(M) (M = Mg(II), Fe(II) and Ni(II)) in the battery cell, the batteries assembled based on S/MOF-74 cathode with different metal ions were analysed. The first three cycles of CV curves at a scan rate of 0.1 mV s^−1^ over the voltage range of 1.8–2.8 V are shown in Fig. 7a. The cathodic scan shows two sharp peaks at 2.00 V and 2.31 V. The peak located at 2.31 V corresponds to the conversion of sulphur S_8_ to higher polysulphides (Li_2_S_8_, Li_2_S_6_, and Li_2_S_4_). A further reduction to lower polysulphides (Li_2_S_2_) and a final reduction product (Li_2_S) forms a peak observed at 2.00 V. The anodic scan shows two overlapping peaks at 2.37 V and 2.45 V, which are associated with the reverse reaction, oxidation of Li_2_S/ Li_2_S_2_ to higher polysulphides and their eventual conversion to S_8_. Peak current intensity reflects the extent and kinetics of sulfur redox reactions. Higher-potential peaks typically involve larger charge transfer and faster reaction kinetics, resulting in higher current. Lower-potential peaks generally represent intermediate or partial reduction/oxidation steps involving soluble polysulfides, which proceed with slower kinetics and lower current intensity. Meanwhile, the current density of S/MOF-74(Fe) was higher than those of S/MOF-74(Ni) and S/MOF-74(Mg). However, the stability of S/MOF-74(Ni) and S/MOF-74(Mg) over three scans is significantly higher than that of S/MOF-74(Fe). This finding may be caused by the increased conductivity of Fe(II) ions and the lower chemisorption ability of polysulphides in MOF-74(Fe). The high chemisorption ability of S/MOF-74(Ni) and S/MOF-74(Mg) may accelerate the electrochemical reaction kinetics.

Constant current charge/discharge tests at different C-rates were applied for the examination of S/MOF-74(Mg), S/MOF-74(Fe) and S/MOF-74(Ni) cathodes (see Fig. 7b). The S/MOF-74(Fe) cathode exhibits an initial specific capacity of 645 mAh g^−1^ at 0.2 C. However, the capacity decreases significantly during the first 5 cycles, and after 20 cycles the capacity decreases to 560 mAh g^−1^. The S/MOF-74(Mg) and S/MOF-74(Ni) cathodes show initial capacities of 646 mAh g^−1^ and 614 mAh g^−1^ at 0.2 C, however, their cycle performance is stable, and the capacity increases after 20 cycles up to 684 mAh g^−1^ and 628 mAh g^−1^, respectively. The capacity of S/MOF-74(Mg), S/MOF-74(Ni), and S/MOF-74(Fe) decreases to 590/519/417 mAh g^−1^ at 0.5 C, 440/443/308 mAh g^−1^ at 1 C, 280/315/192 mAh g^−1^ at 2 C, and is recovered to 656/627/492 mAh g^−1^ at 0.2 C. The capacity at a low C-rate was the highest for S/MOF-74(Mg), nevertheless, the best performance at a high C-rate was for S/MOF-74(Ni) cathode. The improved stability at a high C-rate may benefit from the chemical interaction between Ni(II) ions in MOF-74(Ni) and lithium polysulphides.

The first two cycles at 0.2 C are plotted in Fig. S4 in ESI, where the difference between them is negligible for S/MOF-74(Mg) (d) and S/MOF-74(Ni) (f), but for S/MOF-74(Fe) (e) is more significant where can be assumed more significant loss of active material. Voltage profiles at different C-rates from 0.2 C to 2 C for S/MOF-74(Mg), S/MOF-74(Ni), and S/MOF-74(Fe) electrodes are shown in Fig. 7c–e. The presented cycles are the last cycles from each C-rate, thus cycles 20 (0.2 C), 25 (0.5 C), 30 (1 C), and 35 (2 C). Both plateaus are clearly visible for all electrodes and all C-rates. However, the low voltage plateau for the S/MOF-74(Fe) electrode at 2 C was significantly shifted to a lower potential and shortened. It may be observed due to the weaker chemical interaction of Fe(II) ions with lithium polysulphides than Ni(II) and Mg(II), which was also confirmed by XPS analysis, showing the smallest binding energy shift in the S 2*p* region for the Fe(II)-based composite.

The cycling stability of the S/MOF-74(Mg), S/MOF-74(Ni), and S/MOF-74(Fe) electrodes at 0.5 C is represented in Fig. 7f. The initial discharge specific capacity for the S/MOF-74(Mg), S/MOF-74(Ni), and S/MOF-74(Fe) electrodes was 526, 466, and 511 mAh g^−1^, respectively. The reversible capacity after 200 cycles remains high for the S/MOF-74(Mg) and S/MOF-74(Ni) of 410 and 465 mAh g^−1^, respectively. However, the capacity of the S/MOF-74(Fe) electrode significantly decreased during cycling, which resulted in a very low capacity of 14 mAh g^−1^ after 200 cycles. The S/MOF-74(Ni) showed extremely stable cycling performance with a capacity retention of 99.75%, The capacity retention for the S/MOF-74(Mg) was 77.96%, and S/MOF-74(Fe) showed only 2.78% as the capacity at the end of cycling was almost zero. The excellent cycling performance of the S/MOF-74(Ni) electrode may be attributed to improved sulphur utilization due to the unique structure of MOF-74(Ni) that immobilizes sulphur and strong physical and chemical adsorption of polysulphides thanks to Ni(II) ions. The average Coulombic efficiency for the S/MOF-74(Ni) electrode during 200 cycles at 0.5 C was around 89.38%, and for the S/MOF-74(Mg) electrode was 90.36%. Table 3 compares the electrochemical performance of various sulphur cathodes containing MOF materials in Li–S batteries. It can be observed that the S/MOF-74(Ni) cathode maintains remarkable performance and cycling stability. The adsorption and catalytic transformation of lithium polysulphides was achieved by an extremely stable cycle performance of the S/MOF-74(Ni) electrode.

**Table 3:** Comparison of sulphur cathodes containing MOF in Li–S batteries and their electrochemical properties.

Material structure	C-rate	Cycle number	Initial capacity (mAh g^−1^)	Reversible capacity (mAh g^−1^)	Decay rate per cycle (%)	S loading (mg cm^−2^)	References
ZnCO_2_O_4_@Ti_3_C_2_	0.5 C	400	1142	306	0.183	1.0–1.5	^68^
ZIF-8-P–C	0.2 C	100	1104	561	0.492	2.0	^88^
MWCNT@MOF-5/S	0.5 C	50	874	272	1.380	1.5	^89^
ZIF-67-S	0.1 C	100	1220	422	0.650	–	^90^
S/Fe-MIL-88	0.1 C	80	820	157	1.013	–	^91^
S/HKUST-1	0.5 C	300	431	286	0.112	–	^92^
S/MOF-74(Mg)	0.5 C	200	526	410	0.110	2.7	This work
S/MOF-74(Ni)	0.5 C	200	466	465	0.001	2.7	This work

It could be noted that commercial Li-ion batteries typically deliver specific capacities in the range of 150–250 mAh g^−1^^1,2^, which is significantly lower than the reversible capacities achieved in this work (e.g., 465 mAh g^−1^ for S/MOF-74(Ni) after 200 cycles). This underlines the practical relevance of the presented results, demonstrating that even without maximizing the absolute initial capacity, the designed MOF-74-based electrodes outperform state-of-the-art commercial systems in terms of energy storage capability.

To further investigate the performance of the S/MOF-74 electrodes, asymmetric cycling (charging at 0.5 C and discharging at 1 C) was performed (see Fig. 7g). In an effort to investigate the impact of the discharging current, the charging current was kept the same as for the previous cycling at 0.5 C. The cell’s behaviour was comparable to symmetric cycling at 0.5 C. The cycling performance of S/MOF-74(Ni) and S/MOF-74(Mg) was highly stable, the capacity in the 1st cycle was 514 and 470 mAh g^−1^and in the 100th cycle 543 and 523 mAh g^−1^, respectively. However, the performance on the S/MOF-74(Fe) electrode was unstable and the capacity decay was very fast, the capacity decreased from 516 to 299 mAh g^−1^ during 100 cycles and the capacity retention was only 57.95%.

**Fig. 8:**
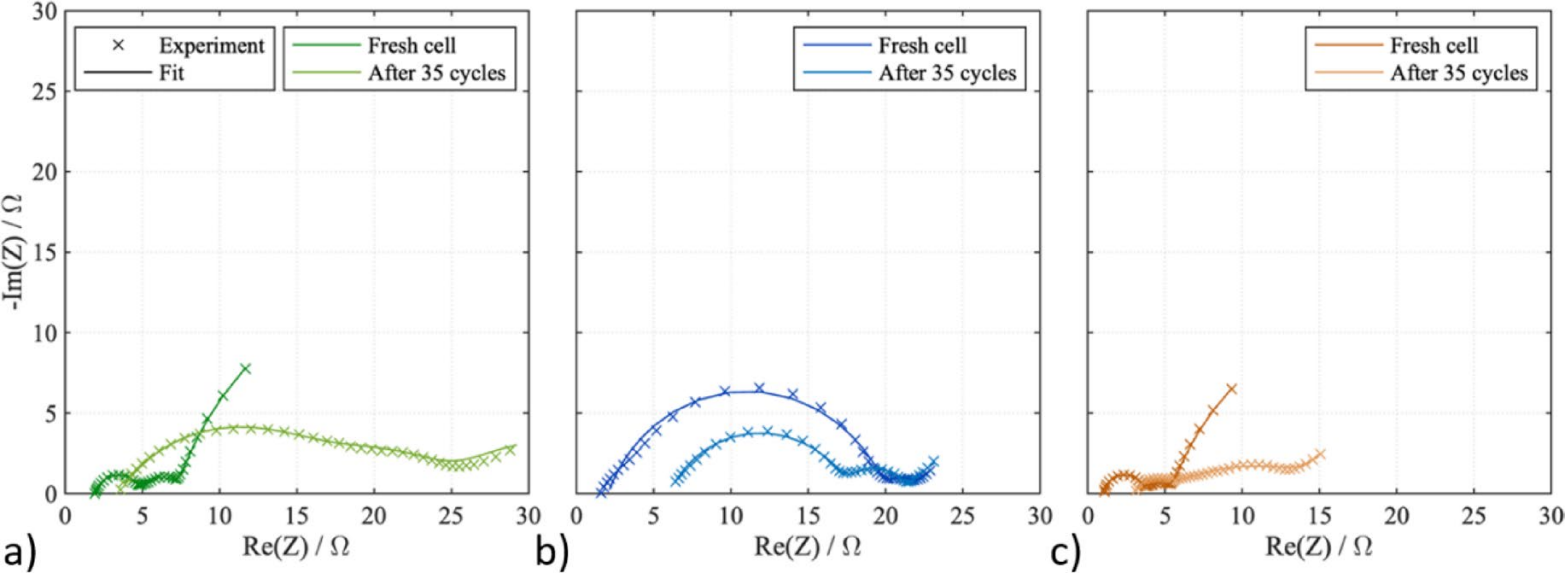
EIS curves before cycling and after 35 cycles for (**a**) S/MOF-74(Mg), (**b**) S/MOF-74(Ni), and (**c**) S/MOF-74(Ni).

The electrochemical performance of S/MOF-74 electrodes was investigated by EIS at the beginning of cycling and after 35 cycles (see Fig. 8). EIS spectra were fitted using the equivalent circuit depicted in Fig. S5 in ESI. The fitted resistance values are summarized in Table 4 where *R*_*e*_ stands for electrolyte resistance, *R*_*ct*_ for charge transfer resistance, *R*_*int*_ for interphase contact resistance, and *R*_*diff*_ for diffusion resistance. The most significant difference between S/MOF-74(M) samples was in the charge transfer resistance. The highest charge transfer resistance of around 18 Ω was for the S/MOF-74(Fe) electrode which may also be a reason for the worst cycle performance of this sample. The charge transfer resistances for S/MOF-74(Ni) and S/MOF-74(Mg) were significantly lower, 2.5 and 3.0 Ω. After 35 cycles, the charge transfer resistance decreased even more for the S/MOF-74(Ni) electrode, suggesting improved contact between particles in the electrode material, which is in agreement with the improved cycle performance and stable cycling.

**Table 4:** The values of resistances for the sulphur-based cathodes containing MOF-74 before cycling and after 35 cycles.

	S/MOF-74(Mg)		S/MOF-74(Fe)		S/MOF-74(Ni)	
	Before cycling	After 35 cycles	Before cycling	After 35 cycles	Before cycling	After 35 cycles
*R*_*e*_ (Ω)	1.9	3.5	2.1	6.0	1.8	3.4
*R*_*ct*_ (Ω)	3.0	15.0	17.8	12.7	2.5	1.8
*R*_*int*_ (Ω)	2.3	8.6	2.2	3.5	1.8	5.9
*R*_*diff*_ (Ω)	37.4	12.8	17.9	11.7	32.6	12.3

In an effort to study the shuttle effect, direct shuttle current measurement was performed at 2 and 10% DOD, as the shuttle effect occurs only in the high voltage plateau where higher polysulfides are present. The correlation between Coulombic efficiency and shuttle current was presented in^6^. The measured shuttle current values are summarized in Table 5. The shuttle current was higher for 2% DOD compared to 10% DOD for all samples. At the beginning of cycling, the lowest shuttle current was for the S/MOF-74(Ni) electrode and the highest was for the S/MOF-74(Fe) electrode, suggesting improved polysulfide confinement for S/MOF-74(Ni). After 100 cycles, the shuttle current increased for all electrodes, probably due to the escalating shuttle effect with the battery degradation, which is in agreement with decreasing Coulombic efficiency. The most significant shuttle current increase was for the S/MOF-74(Fe), as for this electrode material, the continuous capacity decrease was observed. Coulombic efficiency decreases with the cycle number, which is in agreement with an increased shuttle current after cycling. It can be assumed that the shuttle effect is slowly increasing during cycling. The results of the shuttle current measurements are in an agreement with XPS analysis, showing the smallest binding energy shift in the S 2*p* region for the Fe(II)-based composite and the the most significant with the Ni(II)-based framework. In order to visually verify the inhibition of the shuttle effect by MOF-74(M) composites, a visual shuttle test was performed. The yellow solution of Li_2_S_6_ was decolourised after the addition of all MOF-74(M)/C composites. Figure S6 in the ESI shows the Li_2_S_6_ adsorption test of MOF-74(M)/C composites in DOL/DME after 1 h, where a clear solution was observed above the MOF-74(M)-based composites, indicating effective polysulfide adsorption.

**Table 5:** Shuttle current for S/MOF-74 electrodes at 2% and 10% DOD before cycling and after 100 cycles.

DOD (%)	Shuttle current (mA g^−1^)					
	S/MOF-74(Mg)		S/MOF-74(Fe)		S/MOF-74(Ni)	
	Before cycling	After cycling	Before cycling	After cycling	Before cycling	After cycling
2	1.98	2.38	2.08	5.07	1.43	3.20
10	1.07	1.39	1.36	3.70	1.15	1.85

### SEM and EDX analyses

**Fig. 9:**
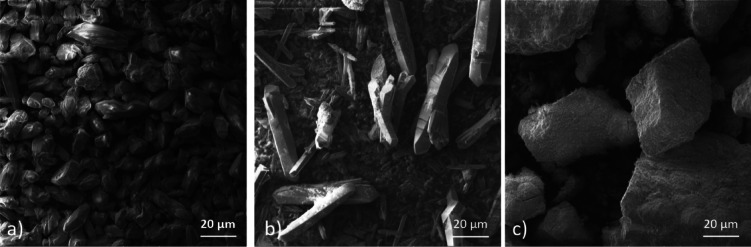
SEM images of (**a**) MOF-74(Mg), (**b**) MOF-74(Fe), and (**c**) MOF-74(Ni).

The particle size and morphology of MOF-74(M) (M = Mg(II), Fe(II) and Ni(II)) were observed by scanning electron microscopy. As shown in Fig. 9c, the grains of MOF-74(Ni) have an irregular shape with a smooth surface and a size of about 40–80 μm. The change of the metal ion in MOF-74 significantly influences the shape of crystals. MOF-74(Mg) (see Fig. 9a) exhibited small block-like crystals in a size range of 10–30 μm. Needle-shaped crystals with a length in a range of 20–80 μm were observed for MOF-74(Fe) (see Fig. 9b).

**Fig. 10:**
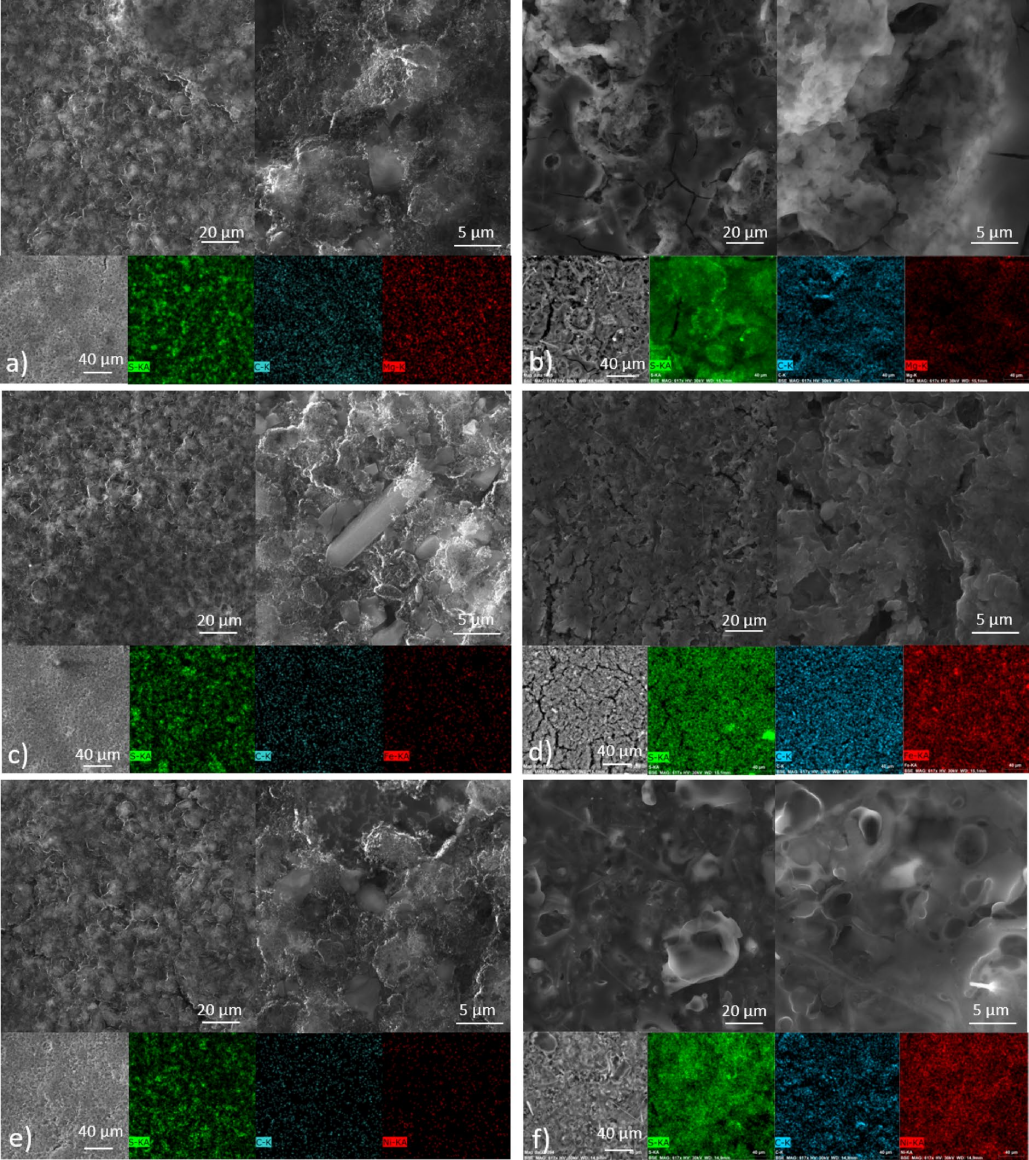
SEM images and elemental maps of sulphur electrodes with MOF-74(Mg), MOF-74(Fe), and MOF-74(Ni) before cycling (**a**, **c**, **e**) and after 100 cycles (**b**, **d**, **f**).

The surface and morphology of the sulphur-based electrodes with MOF-74(M) (M = Mg(II), Fe(II) and Ni(II)) were investigated using SEM and EDX analyses. All electrodes were porous enough for electrolyte penetration, and their surface morphology was comparable. MOF crystals are visible at higher magnification of all pristine electrodes (see Fig. 10a) S/MOF-74(Mg), c) S/MOF-74(Fe) and e) S/MOF-74(Ni)). The sulphur and metal (Ni/Mg/Fe) distribution was uniform for all prepared electrodes without significant clusters, as depicted in Fig. 10. Electrode materials were also analysed after 100 cycles using asymmetric cycling, charging at 0.5 C and discharging at 1 C electrodes (see Fig. 10b) S/MOF-74(Mg), d) S/MOF-74(Fe) and f) S/MOF-74(Ni)). The electrode surface is different after cycling, and dissolved sulphur is visible. The electrode material for S/MOF-74(Ni) and S/MOF-74(Mg) is without any significant cracks, indicating improved accommodation of volumetric changes of sulphur. However, there are several visible cracks in the S/MOF-74(Fe) electrode responsible for worse particle connection, which might be the reason for the rapid capacity decrease during cycling.

### Structure-performance relationships

A deeper understanding of how the physicochemical properties of MOF-74(M) (M = Mg(II), Fe(II) and Ni(II)) materials influence their electrochemical performance as sulfur hosts in Li–S batteries was achieved by correlating structural characterization results with electrochemical data. Although the MOF-74 frameworks with different metal centers (Ni(II), Mg(II), and Fe(II)) are isostructural, they exhibit distinct behavior in terms of thermal stability, porosity, and chemical interaction with sulfur, which ultimately impacts their performance in composite cathodes.

Nitrogen adsorption/desorption measurements revealed that the optimal activation temperature varied depending on the metal center. For MOF-74(Ni) and MOF-74(Mg), increasing the activation temperature led to a substantial rise in surface area, reaching 1315 m^2^ g^−1^ and 1175 m^2^ g^−1^, respectively, at 250 °C. In contrast, MOF-74(Fe) showed the highest surface area (1307 m^2^ g^−1^) at only 60 °C, with degradation occurring at higher temperatures. After sulfur loading, all S/MOF-74 composites showed a drastic reduction in BET surface area (to 9, 17, and 5 m^2^ g^−1^ for Ni(II)-, Mg(II)-, and Fe(II)-based materials, respectively) and a notable increase in apparent pore diameter from ~ 1.0 to 3.1–3.8 nm. These changes indicate successful infiltration of sulfur into the porous structure and partial pore blockage. The reduction in pore volume and surface area correlates well with CHNS analysis, which confirmed high sulfur loading (~ 60 wt%) in all composites.

XPS analysis provided insight into the chemical interactions between sulfur and the MOF-74 frameworks. The S 2*p* binding energy shifts relative to elemental sulfur reflected the strength of interaction with metal centers. The most pronounced shift (ǀΔǀ = 0.66 eV) was observed for S/MOF-74(Ni), followed by S/MOF-74(Mg) (ǀΔǀ = 0.64 eV), and the lowest for S/MOF-74(Fe) (ǀΔǀ = 0.59 eV). These results suggest that the Ni(II)-based framework has the strongest interaction with sulfur species, which can improve sulfur immobilization and suppress polysulfide diffusion during cycling. These trends were reflected in the electrochemical behavior of the composites.

PXRD patterns of the S/MOF-74 materials showed that the framework remained crystalline. FTIR spectroscopy also confirmed the presence of carboxylate-related vibrations, further supporting the structural integrity of the MOF after electrode fabrication. Post-mortem SEM analysis revealed that the electrodes containing S/MOF-74(Ni) and S/MOF-74(Mg) remained compact and free of significant cracks after 100 cycles, while S/MOF-74(Fe) showed visible structural damage and surface cracking, indicating mechanical degradation and poor particle connectivity.

These structural findings align well with the electrochemical results. S/MOF-74(Ni) demonstrated the best performance, including high specific capacity, excellent capacity retention (99.75% after 200 cycles at 0.5 C), low charge transfer resistance, and minimal shuttle current. The superior performance is attributed to its high porosity, strong sulfur–metal interactions, thermal stability, and mechanical robustness. The superior electrochemical stability of S/MOF-74(Ni), and to a lesser extent S/MOF-74(Mg), can be rationalized by the encapsulation mechanism of sulfur within the MOF-74 framework. On the one hand, the hexagonal channels of MOF-74 (~ 11 Å) physically confine S₈ molecules (~ 4.5 Å), which is directly evidenced by the drastic reduction in surface area and pore volume after sulfur incorporation (Table 1, Fig. 2d). On the other hand, the coordinatively unsaturated metal sites (CUSs) provide Lewis acidic centers that interact with sulfur species, as confirmed by the binding energy shifts observed in the S 2*p* XPS spectra (Fig. 5). This dual mechanism, micropore confinement and Lewis acid–base interactions, explains the effective sulfur immobilization and suppression of the polysulfide shuttle, thereby establishing a direct link between the framework structure and the enhanced electrochemical performance. In contrast, S/MOF-74(Fe) showed the weakest performance, consistent with its poor stability, low surface area after activation, weak sulfur interaction, and observed electrode degradation.

In conclusion, the electrochemical performance of S/MOF-74 cathodes is closely governed by the synergy of structural factors such as accessible surface area, pore architecture, sulfur–framework interaction strength, and post-cycling morphological integrity. These findings highlight the critical importance of optimizing the MOF structure and activation conditions to enhance sulfur confinement and long-term cycling stability in Li–S batteries.

## Conclusions

In summary, MOF-74(M) (M = Mg(II), Fe(II) and Ni(II)) were synthesized, characterized (nitrogen adsorption/desorption, XPS, PXRD, FTIR, TGA, and SEM analyses) and used as a functional host for sulphur in Li–S batteries. Different metal ions in the MOF-74 platform and their effect on the performance of the Li–S battery cell were investigated. The S/MOF-74(Ni) showed electrochemical promotion of redox kinetics of lithium polysulphides and their strong adsorption. The porous architecture of MOF-74(Ni) can efficiently confine and encapsulate sulphur, accommodate volumetric expansion during cycling, and inhibit diffusion of lithium polysulphides to ensure long cycle life. The S/MOF-74(Ni) cathode exhibits a capacity of 614 mAh g^−1^ at 0.2 C, 315 mAh g^−1^ at 2 C and superior cycling stability over 200 cycles at 0.5 C with a capacity decay of only 0.001% per cycle. The excellent electrochemical performance could be attributed to the unique porous structure of MOF-74 and the high catalytic properties of Ni(II) ions. The S/MOF-74(Mg) cathode showed decent electrochemical performance with a capacity of 526 mAh g^−1^ at 0.2 C. However, the stability at high current was lower, the capacity reached a value of 280 mAh g^−1^, and the cycling stability over 200 cycles at 0.5 C was 0.110%. The S/MOF-74(Fe) electrode exhibited the poorest cycling stability over long-term cycling at 0.5 C of the selected MOF-74 materials. The shuttle current measurement confirmed the cycling results, as the highest shuttle current was measured for the S/MOF-74(Fe). Moreover, the charge transfer resistance is the lowest for S/MOF-74(Ni) and the highest for S/MOF-74(Fe). The electrochemical results are in an agreement with XPS analysis, showing and the strongest interaction with the Ni(II)-based framework and the weakest for the Fe(II)-based composite.

Our electrochemical investigations including different metal ions in the MOF-74 platform provide supporting guidelines for future selection of MOFs playing the role of porous hosts in Li–S batteries.

## Supplementary Information

Below is the link to the electronic supplementary material.

Supplementary Material 1

## Data Availability

The datasets used and/or analysed during the current study available from the corresponding author on reasonable request.

## References

[1] Mohammadi, F. & Saif, M. A comprehensive overview of the electric vehicle batteries market. *e-Prime* **3**, 100127. 10.1016/j.prime.2023.100127 (2023).

[2] Olabi, A. G., Abbas, Q., Shinde, P. A. & Abdelkareem, M. A. Rechargeable batteries: Technological advancement, challenges, current and emerging applications. *Energy* **266**, 126408. 10.1016/j.energy.2022.126408 (2023).

[3] Gonçalves, J. M., Santos, É. A., Martins, P. R., Silva, C. G. & Zanin, H. Emerging medium- and high-entropy materials as catalysts for lithium-sulfur batteries. *Energy Storage Mater.* **63**, 102999. 10.1016/j.ensm.2023.102999 (2023).

[4] Király, N. et al. Sr(II) and Ba(II) alkaline earth metal–organic frameworks (AE-MOFs) for selective gas adsorption, energy storage, and environmental application. *Nanomaterials* **13**(2), 234. 10.3390/nano13020234 (2023). 36677987 10.3390/nano13020234 PMC9866501

[5] Király, N. et al. Post-synthetically modified metal–porphyrin framework GaTCPP for carbon dioxide adsorption and energy storage in Li–S batteries. *RSC Adv.* **12**(37), 23989–24002. 10.1039/d2ra03301a (2022). 36093251 10.1039/d2ra03301a PMC9400624

[6] Capková, D., Knap, V., Fedorková, A. S. & Stroe, D. Investigation of the temperature and DOD effect on the performance-degradation behavior of lithium–sulfur pouch cells during calendar aging. *Appl. Energy* **332**, 120543. 10.1016/j.apenergy.2022.120543 (2023).

[7] Wang, C. et al. Promoted polysulfide conversion process and improved rate performance by tin atom modified carbon in Li–S batteries. *Appl. Surf. Sci.* **652**, 159283. 10.1016/j.apsusc.2023.159283 (2024).

[8] Capková, D. et al. Influence of metal-organic framework MOF-76(Gd) activation/carbonization on the cycle performance stability in Li–S battery. *J. Energy Storage* **51**, 104419. 10.1016/j.est.2022.104419 (2022).

[9] Capková, D. et al. Activated and carbonized metal-organic frameworks for improved cycle performance of cathode material in lithium-sulphur batteries. *J. Phys.* **2382**(1), 012010. 10.1088/1742-6596/2382/1/012010 (2022).

[10] Li, S. & Fan, Z. Encapsulation methods of sulfur particles for lithium-sulfur batteries: A review. *Energy Storage Mater.* **34**, 107–127. 10.1016/j.ensm.2020.09.005 (2021).

[11] Wang, Z. et al. Single-atomic Co-B_2_N_2_ sites anchored on carbon nanotube arrays promote lithium polysulfide conversion in lithium–sulfur batteries. *Carbon Energy*10.1002/cey2.306 (2023).

[12] Lama, F. L., Marangon, V., Caballero, Á., Morales, J. & Hassoun, J. Diffusional features of a Lithium–Sulfur battery exploiting highly microporous activated carbon. *ChemSusChem*10.1002/cssc.202202095 (2023). 36562306 10.1002/cssc.202202095

[13] Huang, Z. et al. Coordinating interface polymerization with micelle mediated assembly towards two-dimensional mesoporous carbon/coni for advanced lithium–sulfur battery. *Small*10.1002/smll.202207411 (2023). 36965086 10.1002/smll.202207411

[14] Yuan, Y. et al. Nano VS4 in-situ grown in three-dimensional honeycomb macroporous carbon enabling high-rate and long-life lithium storage. *J. Alloys Compd.* **942**, 169021. 10.1016/j.jallcom.2023.169021 (2023).

[15] Fan, C. et al. Graphene quantum dots as sulfiphilic and lithiophilic mediator toward high stability and durable life lithium-sulfur batteries. *J. Energy Chem.* **85**, 254–266. 10.1016/j.jechem.2023.06.030 (2023).

[16] Sun, R. et al. Enhancing polysulfide confinement and electrochemical kinetics by amorphous cobalt phosphide for highly efficient Lithium-Sulfur batteries. *ACS Nano* **15**(1), 739–750. 10.1021/acsnano.0c07038 (2020). 33370111 10.1021/acsnano.0c07038

[17] Pang, Q., Kundu, D., Cuisinier, M. & Nazar, L. F. Surface-enhanced redox chemistry of polysulphides on a metallic and polar host for lithium-sulphur batteries. *Nat. Commun.*10.1038/ncomms5759 (2014). 25154399 10.1038/ncomms5759

[18] Zheng, M. et al. CO/COS2 heterojunction embedded in N, S-doped hollow nanocage for enhanced polysulfides conversion in high-performance lithium–sulfur batteries. *Small.*10.1002/smll.202303192 (2023). 37712177 10.1002/smll.202303192

[19] Zhang, K. et al. High loading sulfur cathodes by reactive-type polymer tubes for high-performance lithium–sulfur batteries. *Adv. Funct. Mater.*10.1002/adfm.202212759 (2022).36714167

[20] Geng, C. et al. Demystifying the catalysis in lithium–sulfur batteries: Characterization methods and techniques. *SusMat* **1**(1), 51–65. 10.1002/sus2.5 (2021).

[21] Huang, J. et al. MOF-based materials for electrochemical reduction of carbon dioxide. *Coord. Chem. Rev.* **494**, 215333. 10.1016/j.ccr.2023.215333 (2023).

[22] Obeso, J. L. et al. The role of dynamic metal-ligand bonds in metal-organic framework chemistry. *Coord. Chem. Rev.* **496**, 215403. 10.1016/j.ccr.2023.215403 (2023).

[23] Ma, D. et al. Multifunctional nano MOF drug delivery platform in combination therapy. *Eur. J. Med. Chem.* **261**, 115884. 10.1016/j.ejmech.2023.115884 (2023). 37862817 10.1016/j.ejmech.2023.115884

[24] Almáši, M. A review on state of art and perspectives of metal-organic frameworks (MOFs) in the fight against coronavirus SARS-CoV-2. *J. Coord. Chem.* **74**(13), 2111–2127. 10.1080/00958972.2021.1965130 (2021).

[25] Chen, G. et al. Zeolites and metal–organic frameworks for gas separation: The possibility of translating adsorbents into membranes. *Chem. Soc. Rev.* **52**(14), 4586–4602. 10.1039/d3cs00370a (2023). 37377411 10.1039/d3cs00370a

[26] Zeleňák, V., Vargová, Z., Almáši, M., Kuchár, J. & Zeleňáková, A. Layer-pillared zinc(II) metal-organic framework built from 4,4′-azo(bis)pyridine and 1,4-BDC. *Microporous Mesoporous Mater.* **129**(3), 354–359. 10.1016/j.micromeso.2009.11.002 (2010).

[27] Zelenka, T. et al. Carbon dioxide and hydrogen adsorption study on surface-modified HKUST-1 with diamine/triamine. *Sci. Rep.*10.1038/s41598-022-22273-2 (2022). 36253389 10.1038/s41598-022-22273-2 PMC9574841

[28] Hossain, S. S., Karthik, V., Dhakshinamoorthy, A. & Biswas, S. A recyclable MOF@polymer thin film composite for nanomolar on-site fluorometric detection of heavy metal ion and anti-histamine drug and efficient heterogeneous catalysis of Friedel-Crafts alkylation. *Inorg. Chem. Front.* **11**(1), 142–155. 10.1039/d3qi01890c (2024).

[29] Zeleňák, V. et al. Large and tunable magnetocaloric effect in gadolinium-organic framework: tuning by solvent exchange. *Sci. Rep.* **9**(11), 15572. 10.1038/s41598-019-51590-2 (2019). 31666558 10.1038/s41598-019-51590-2 PMC6821888

[30] Szufla, M., Navarro, J. A. R., Góra-Marek, K. & Matoga, D. Effect of Missing-Linker defects and ion exchange on stability and proton conduction of a sulfonated layered Zr-MOF. *ACS Appl. Mater. Interfaces* **15**(23), 28184–28192. 10.1021/acsami.3c03873 (2023). 37265204 10.1021/acsami.3c03873 PMC10273224

[31] Almáši, M., Sharma, A. & Zelenka, T. Anionic zinc(II) metal-organic framework post-synthetically modified by alkali-ion exchange: Synthesis, characterization and hydrogen adsorption properties. *Inorg. Chim. Acta* **526**, 120505. 10.1016/j.ica.2021.120505 (2021).

[32] Sohrabi, H. et al. Metal-organic frameworks (MOF)-based sensors for detection of toxic gases: A review of current status and future prospects. *Mater. Chem. Phys.* **299**, 127512. 10.1016/j.matchemphys.2023.127512 (2023).

[33] Garg, A. et al. A highly stable terbium(III) metal-organic framework MOF-76(Tb) for hydrogen storage and humidity sensing. *Environ. Sci. Pollut. Res.* **30**(44), 98548–98562. 10.1007/s11356-022-21290-y (2022). 10.1007/s11356-022-21290-y 35688971

[34] Garg, A. et al. Gd(III) metal-organic framework as an effective humidity sensor and its hydrogen adsorption properties. *Chemosphere* **305**, 135467. 10.1016/j.chemosphere.2022.135467 (2022). 35764119 10.1016/j.chemosphere.2022.135467

[35] Geng, P. et al. MIL-96-Al for Li–S batteries: Shape or size?. *Adv. Mater.*10.1002/adma.202107836 (2021). 34719819 10.1002/adma.202107836

[36] Li, W. et al. Rational design and general synthesis of multimetallic metal–organic framework nano-octahedra for enhanced Li–S battery. *Adv. Mater.*10.1002/adma.202105163 (2021). 34554610 10.1002/adma.202105163

[37] Zhou, J. et al. Synthesis of S/CoS_2_ nanoparticles-embedded n-doped carbon polyhedrons from polyhedrons ZIF-67 and their properties in lithium–sulfur batteries. *Electrochim. Acta* **218**, 243–251. 10.1016/j.electacta.2016.09.130 (2016).

[38] Yang, Y. et al. A heterogenized Ni-doped zeolitic imidazolate framework to guide efficient trapping and catalytic conversion of polysulfides for greatly improved lithium–sulfur batteries. *J. Mater. Chem. A Mater. Energy Sustain.* **6**(28), 13593–13598. 10.1039/c8ta05176c (2018).

[39] Zhang, X. et al. Enhanced cycling performance of rechargeable Li–O_2_ batteries via LiOH formation and decomposition using high-performance MOF-74@CNTs hybrid catalysts. *Energy Storage Mater.* **17**, 167–177. 10.1016/j.ensm.2018.11.014 (2019).

[40] Yan, W. et al. Downsizing metal–organic frameworks with distinct morphologies as cathode materials for high-capacity Li–O_2_ batteries. *Mater. Chem. Front.* **1**(7), 1324–1330. 10.1039/c6qm00338a (2017).

[41] Park, H. & Siegel, D. J. Tuning the adsorption of polysulfides in lithium–sulfur batteries with metal–organic frameworks. *Chem. Mater.* **29**(11), 4932–4939. 10.1021/acs.chemmater.7b01166 (2017).

[42] Zhang, Z. et al. Hexagonal rodlike CU-MOF-74-derived filler-reinforced composite polymer electrolyte for high-performance solid-state lithium batteries. *ACS Appl. Energy Mater.* **5**(1), 1095–1105. 10.1021/acsaem.1c03462 (2022).

[43] Sung, S. H., Kim, B. H., Lee, S. T., Choi, S. C. & Yoon, W. Y. Increasing sulfur utilization in lithium-sulfur batteries by a Co-MOF-74@MWCNT interlayer. *J. Energy Chem.* **60**, 186–193. 10.1016/j.jechem.2020.12.033 (2021).

[44] Wu, H., Zhou, W. & Yildirim, T. High-capacity methane storage in metal−organic frameworks M2(dhtp): The important role of open metal sites. *J. Am. Chem. Soc.* **131**(13), 4995–5000. 10.1021/ja900258t (2009). 19275154 10.1021/ja900258t

[45] Kim, H. & Hong, C. S. MOF-74-type frameworks: Tunable pore environment and functionality through metal and ligand modification. *CrystEngComm* **23**(6), 1377–1387. 10.1039/d0ce01870h (2021).

[46] Kim, D. & Coskun, A. Template-directed approach towards the realization of ordered heterogeneity in bimetallic metal–organic frameworks. *Angew. Chem. Int. Ed.* **56**(18), 5071–5076. 10.1002/anie.201702501 (2017). 10.1002/anie.201702501 28370921

[47] Zhang, Z. et al. Modulating the basicity of Zn-MOF-74 via cation exchange with calcium ions. *Dalton Trans.* **48**(40), 14971–14974. 10.1039/c9dt03332g (2019). 31559976 10.1039/c9dt03332g

[48] Wang, L. J. et al. Synthesis and characterization of metal–organic framework-74 containing 2, 4, 6, 8, and 10 different metals. *Inorg. Chem.* **53**(12), 5881–5883. 10.1021/ic500434a (2014). 24878113 10.1021/ic500434a

[49] Choi, I. et al. Facile synthesis of M-MOF-74 (M=Co, Ni, Zn) and its application as an ElectroCatalyst for electrochemical CO_2_ conversion and H_2_ production. *J. Electrochem. Sci. Technol.* **8**(1), 61–68. 10.5229/jecst.2017.8.1.61 (2017).

[50] Garg, A. et al. Metal-organic framework MOF-76(Nd): Synthesis, characterization, and study of hydrogen storage and humidity sensing. *Front. Energy Res.* **8**, 604735. 10.3389/fenrg.2020.604735 (2021).

[51] Beamish-Cook, J., Shankland, K., Murray, C. A. & Vaqueiro, P. Insights into the mechanochemical synthesis of MOF-74. *Cryst. Growth Des.* **21**(5), 3047–3055. 10.1021/acs.cgd.1c00213 (2021). 34267598 10.1021/acs.cgd.1c00213 PMC8273859

[52] Ling, J. et al. One-pot method synthesis of bimetallic MgCu-MOF-74 and its CO_2_ adsorption under visible light. *ACS Omega* **7**(23), 19920–19929. 10.1021/acsomega.2c01717 (2022). 35722001 10.1021/acsomega.2c01717 PMC9202246

[53] Chen, C., Kosari, M., Jing, M. & He, C. Microwave-assisted synthesis of bimetallic NiCo-MOF-74 with enhanced open metal site for efficient CO_2_ capture. *Environ. Funct. Mater.* **1**(3), 253–266. 10.1016/j.efmat.2023.01.002 (2022).

[54] Wauteraerts, N. et al. Vapor-assisted synthesis of the MOF-74 metal–organic framework family from zinc, cobalt, and magnesium oxides. *Dalton Trans.* **52**(47), 17873–17880. 10.1039/d3dt01785k (2023). 37975724 10.1039/d3dt01785k

[55] Das, A. K., Vemuri, R. S., Kutnyakov, I., McGrail, B. P. & Motkuri, R. K. An efficient synthesis strategy for metal-organic frameworks: Dry-gel synthesis of MOF-74 framework with high yield and improved performance. *Sci. Rep.*10.1038/srep28050 (2016). 27306598 10.1038/srep28050 PMC4910056

[56] Rettig, S. J. & Trotter, J. Refinement of the structure of orthorhombic sulfur, α-S8. *Acta Crystallogr. C* **43**(12), 2260–2262. 10.1107/s0108270187088152 (1987).

[57] Liu, G., Qin, Y., Jing, L., Wei, G. & Li, H. Two novel MOF-74 analogs exhibiting unique luminescent selectivity. *Chem. Commun.* **49**(17), 1699–1701. 10.1039/c2cc37140e (2012). 10.1039/c2cc37140e 23212209

[58] Nishi, Y. The dawn of lithium-ion batteries. *Electrochem. Soc. Interface* **25**(3), 71–74. 10.1149/2.f06163if (2016).

[59] Hong, K. The development of hydrogen storage electrode alloys for nickel hydride batteries. *J. Power Sources* **96**(1), 85–89. 10.1016/s0378-7753(00)00678-9 (2001).

[60] Jung, R., Metzger, M., Maglia, F., Stinner, C. & Gasteiger, H. A. Oxygen release and its effect on the cycling stability of LiNiXMNYCOZO2(NMC) cathode materials for Li-Ion batteries. *J. Electrochem. Soc.* **164**(7), A1361–A1377. 10.1149/2.0021707jes (2017).

[61] Caskey, S. R., Wong-Foy, A. G. & Matzger, A. J. Dramatic tuning of carbon dioxide uptake via metal substitution in a coordination polymer with cylindrical pores. *J. Am. Chem. Soc.* **130**(33), 10870–10871. 10.1021/ja8036096 (2008). 18661979 10.1021/ja8036096

[62] Bhattacharjee, S. et al. Solvothermal synthesis of FE-MOF-74 and its catalytic properties in phenol hydroxylation. *J. Nanosci. Nanotechnol.* **10**(1), 135–141. 10.1166/jnn.2010.1493 (2010). 20352823 10.1166/jnn.2010.1493

[63] Zauška, Ľ et al. Tuning the photocatalytic performance of mesoporous silica-titanium dioxide and cobalt titanate for methylene blue and Congo red adsorption/photodegradation: Impact of azo dyes concentration, catalyst mass, wavelength, reusability and kinetic properties. *J. Photochem. Photobiol. Chem.*10.1016/j.jphotochem.2024.115522 (2024).

[64] Zelenka, T. et al. The influence of HKUST-1 and MOF-76 hand grinding/mechanical activation on stability, particle size, textural properties and carbon dioxide sorption. *Sci. Rep.*10.1038/s41598-024-66432-z (2024). 38965298 10.1038/s41598-024-66432-z PMC11224341

[65] Moy, D., Manivannan, A. & Narayanan, S. R. Direct measurement of polysulfide shuttle current: A window into understanding the performance of lithium–sulfur cells. *J. Electrochem. Soc.*10.1149/2.0181501jes (2014).

[66] Liang, X. et al. The activation of Co-MOF-74 with open metal sites and their corresponding CO/N_2_ adsorptive separation performance. *Microporous Mesoporous Mater.* **320**, 111109. 10.1016/j.micromeso.2021.111109 (2021).

[67] Ayoub, G. et al. Rational synthesis of mixed-metal microporous metal–organic frameworks with controlled composition using mechanochemistry. *Chem. Mater.* **31**(15), 5494–5501. 10.1021/acs.chemmater.9b01068 (2019).

[68] Wei, A., Wang, L. & Li, Z. Metal-organic framework derived binary-metal oxide/MXene composite as sulfur host for high-performance lithium-sulfur batteries. *J. Alloys Compd.* **899**, 163369. 10.1016/j.jallcom.2021.163369 (2022).

[69] De Freitas Filho, R. L. et al. Influence of the amount of sulfur supported on sustainable ordered mesoporous carbons from tannin for high-performance electrodes in lithium-sulfur batteries. *Microporous Mesoporous Mater.*10.1016/j.micromeso.2025.113530 (2025).

[70] Niščáková, V. et al. Novel Cu(II)-based metal–organic framework STAM-1 as a sulfur host for Li–S batteries. *Sci. Rep.*10.1038/s41598-024-59600-8 (2024). 38649384 10.1038/s41598-024-59600-8 PMC11035644

[71] Niščáková, V. et al. Investigation of polypyrrole based composite material for lithium sulfur batteries. *Sci. Rep.*10.1038/s41598-024-74119-8 (2024). 39358464 10.1038/s41598-024-74119-8 PMC11446934

[72] Quintero-Álvarez, F. G. et al. Lanthanide-based metal-organic frameworks MOF-76 for the depollution of xenobiotics from water: Arsenic and fluoride adsorption properties and multi-anionic mechanism analysis. *J. Mol. Struct.* **133825**, 142113. 10.1016/j.molstruc.2025.142113 (2025).

[73] Hu, J., Chen, Y., Zhang, H. & Chen, Z. Controlled syntheses of Mg-MOF-74 nanorods for drug delivery. *J. Solid State Chem.* **294**, 121853. 10.1016/j.jssc.2020.121853 (2020).

[74] Xie, S. et al. MOF-74-M (M = MN Co, NI, ZN, MNCO, MNNI, and MNZN) for low-temperature NH3-SCR and in situ DRIFTS study reaction mechanism. *ACS Appl. Mater. Interfaces* **12**(43), 48476–48485. 10.1021/acsami.0c11035 (2020). 33048536 10.1021/acsami.0c11035

[75] Thommes, M. et al. Physisorption of gases, with special reference to the evaluation of surface area and pore size distribution (IUPAC Technical Report). *Pure Appl. Chem.* **87**(9–10), 1051–1069. 10.1515/pac-2014-1117 (2015).

[76] Lan, Y. & Butler, E. C. Iron-sulfide-associated products formed during reductive dechlorination of carbon tetrachloride. *Environ. Sci. Technol.* **50**(11), 5489–5497. 10.1021/acs.est.5b06154 (2016). 27138348 10.1021/acs.est.5b06154

[77] Wang, W., Xu, R., Yu, B., Wang, X. & Feng, S. Electrochemical fabrication of FeSxfilms with high catalytic activity for oxygen evolution. *RSC Adv.* **9**(55), 31979–31987. 10.1039/c9ra05343c (2019). 35530807 10.1039/c9ra05343c PMC9072975

[78] Liang, X. et al. A highly efficient polysulfide mediator for lithium–sulfur batteries. *Nat. Commun.*10.1038/ncomms6682 (2015). 25562485 10.1038/ncomms6682

[79] Strauss, I. et al. Metal–organic framework Co-MOF-74-based host-guest composites for resistive gas sensing. *ACS Appl. Mater. Interfaces* **11**(15), 14175–14181. 10.1021/acsami.8b22002 (2019). 30900448 10.1021/acsami.8b22002 PMC6492948

[80] Wang, Y. et al. MXENES for sulfur-based batteries. *Adv. Energy Mater.* **13**, 4. 10.1002/aenm.202202860 (2022).

[81] Gao, T. et al. Reversible S0/MGSX redox chemistry in a MGTFSI2/MGCL2/DME electrolyte for rechargeable MG/S batteries. *Angew. Chem. Int. Ed.* **56**(43), 13526–13530. 10.1002/anie.201708241 (2017). 10.1002/anie.201708241 28849616

[82] Xing, J. et al. In situ growth of well-ordered NiFe-MOF-74 on Ni foam by Fe^2+^ induction as an efficient and stable electrocatalyst for water oxidation. *Chem. Commun.* **54**(51), 7046–7049. 10.1039/c8cc03112f (2018). 10.1039/c8cc03112f 29873360

[83] Sun, J., Zhang, X., Zhang, A. & Liao, C. Preparation of Fe–Co based MOF-74 and its effective adsorption of arsenic from aqueous solution. *J. Environ. Sci.* **80**, 197–207. 10.1016/j.jes.2018.12.013 (2018). 10.1016/j.jes.2018.12.013 30952337

[84] Iqbal, R. et al. Exploring the synergistic effect of novel NI-FE in 2D bimetallic metal–organic frameworks for enhanced electrochemical reduction of CO_2_. *Adv. Mater. Interfaces*10.1002/admi.202101505 (2021).

[85] Tan, J., Wu, J., Zhao, J., Xie, L. & Li, G. Highly dispersed ultrafine Ni particles embedded into MOF-74 arrays by partial carbonization for highly efficient hydrogen evolution. *Mater. Adv.* **1**(5), 1212–1219. 10.1039/d0ma00253d (2020).

[86] Fan, R., Kang, N., Li, Y. & Gao, L. A template-directed synthesis of metal–organic framework (MOF-74) ultrathin nanosheets for oxygen reduction electrocatalysis. *RSC Adv.* **11**(16), 9353–9360. 10.1039/d0ra09973b (2021). 35423442 10.1039/d0ra09973b PMC8695273

[87] Su, X. et al. Postsynthetic functionalization of Mg-MOF-74 with tetraethylenepentamine: Structural characterization and enhanced CO_2_ adsorption. *ACS Appl. Mater. Interfaces.* **9**(12), 11299–11306. 10.1021/acsami.7b02471 (2017). 28244732 10.1021/acsami.7b02471

[88] Jiang, Y. et al. Monoclinic ZIF-8 nanosheet-derived 2D carbon nanosheets as sulfur immobilizer for high-performance lithium sulfur batteries. *ACS Appl. Mater. Interfaces.* **9**(30), 25239–25249. 10.1021/acsami.7b04432 (2017). 28686010 10.1021/acsami.7b04432

[89] Bao, W., Zhang, Z., Zhou, C., Lai, Y. & Li, J. Multi-walled carbon nanotubes @ mesoporous carbon hybrid nanocomposites from carbonized multi-walled carbon nanotubes @ metal–organic framework for lithium sulfur battery. *J. Power Sources* **248**, 570–576. 10.1016/j.jpowsour.2013.09.132 (2014).

[90] Ge, X., Li, C., Li, Z. & Yin, L. Tannic acid tuned metal-organic framework as a high-efficiency chemical anchor of polysulfide for lithium-sulfur batteries. *Electrochim. Acta* **281**, 700–709. 10.1016/j.electacta.2018.06.010 (2018).

[91] Yao, J. et al. Reduced graphene oxide coated Fe-soc as a cathode material for high-performance lithium-sulfur batteries. *Ceram. Int.* **46**(15), 24155–24161. 10.1016/j.ceramint.2020.06.195 (2020).

[92] Zhou, J. et al. Rational design of a metal–organic framework host for sulfur storage in fast, long-cycle Li–S batteries. *Energy Environ. Sci.* **7**(8), 2715. 10.1039/c4ee01382d (2014).

