# Supplementary material for: MOF-74(M) (M = Mg(II), Fe(II), Ni(II)) frameworks to enable accelerated redox kinetics for Li–S batteries
Source: Sci Rep. 2025 Nov 3;15:38396. doi: 10.1038/s41598-025-22340-4 (PMC12583643; doi:10.1038/s41598-025-22340-4)
Supplement: Supplementary file 1 — Supplementary Material 1 [file 41598_2025_22340_MOESM1_ESM.docx]

**ELECTRONIC SUPPLEMENTARY INFORMATION**

**(ESI)**

**MOF-74(M) (M = Mg(II), Fe(II), Ni(II)) Frameworks to Enable Accelerated Redox Kinetics for Li-S Batteries**

D. Capková^a,b^, T. Kazda^b^, N. Király^c^, D. Volavka^d^, P. Obšatník^c^, A. Šimek^b^, P. Čudek^b^, D. Matoga^e^, J. Bednarčík^d,f^, A. Straková Fedorková^g^, V. Kuchárová^f^, V. Hornebecq^h^, K.M. Ryan^a^, M. Almáši^c*^

^a^ Department of Chemical Sciences, Bernal Institute, University of Limerick, V94 T9PX Limerick, Ireland

^b^ Department of Electrical and Electronic Technology, Faculty of Electrical Engineering and

Communication, Brno University of Technology, Technická 10, CZ-616 00, Brno, Czech Republic

^c^ Department of Inorganic Chemistry, Faculty of Sciences, Pavol Jozef Šafárik University in Košice,

Moyzesova 11, SK-040 01, Košice, Slovak Republic

^d^ Department of Solid State Physics, Faculty of Sciences, Pavol Jozef Šafárik University in Košice,

Park Angelinum 9, SK-040 01, Košice, Slovak Republic

^e^ Faculty of Chemistry, Jagiellonian University, Gronostajowa 2, PL-30-387 Krakow, Poland

^f^ Institute of Experimental Physics, Slovak Academy of Sciences, Watsonova 47, Kosice SK-040 01 Slovak Republic

^g^ Department of Physical Chemistry, Faculty of Sciences, Pavol Jozef Šafárik University in Košice,

Moyzesova 11, SK-040 01, Košice, Slovak Republic

^h^ Aix-Marseille University, CNRS, MADIREL, F-133 97 Marseille, France

*miroslav.almasi@upjs.sk

**FIGURES**

a)
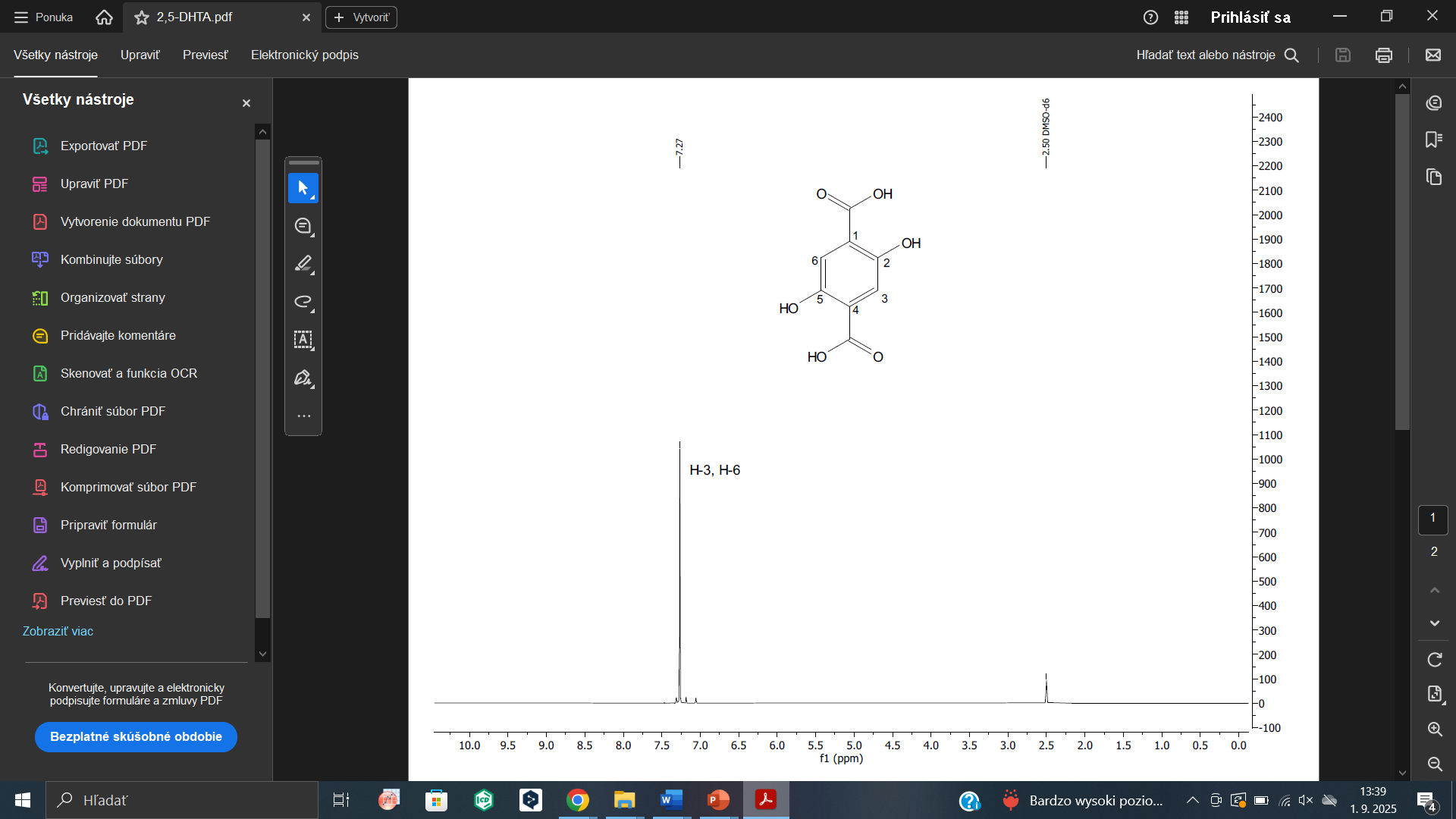


b)
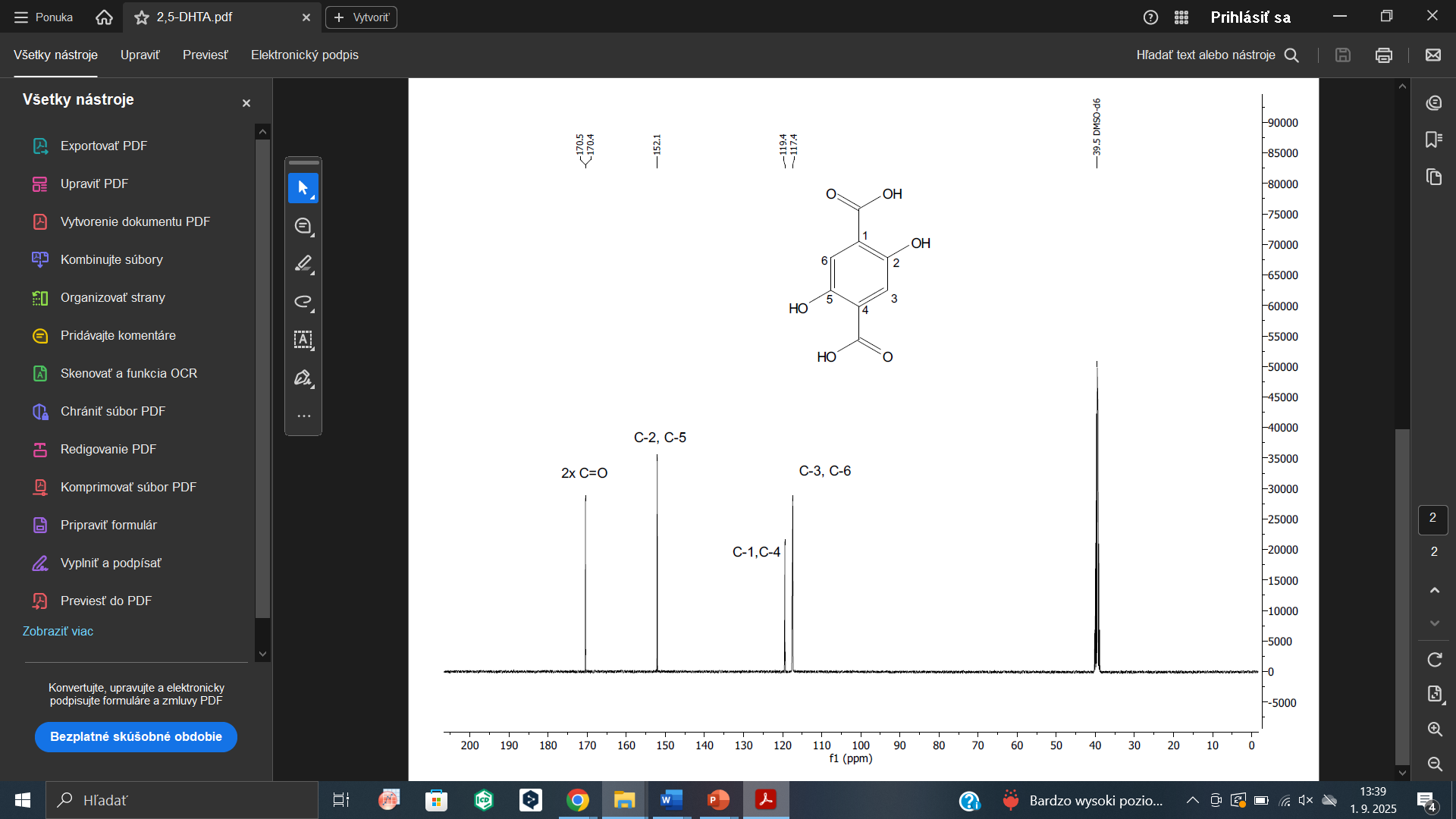


**Fig. S1** a) ^1^H and b) ^13^C NMR spectra of H_4_DOBDC acid.


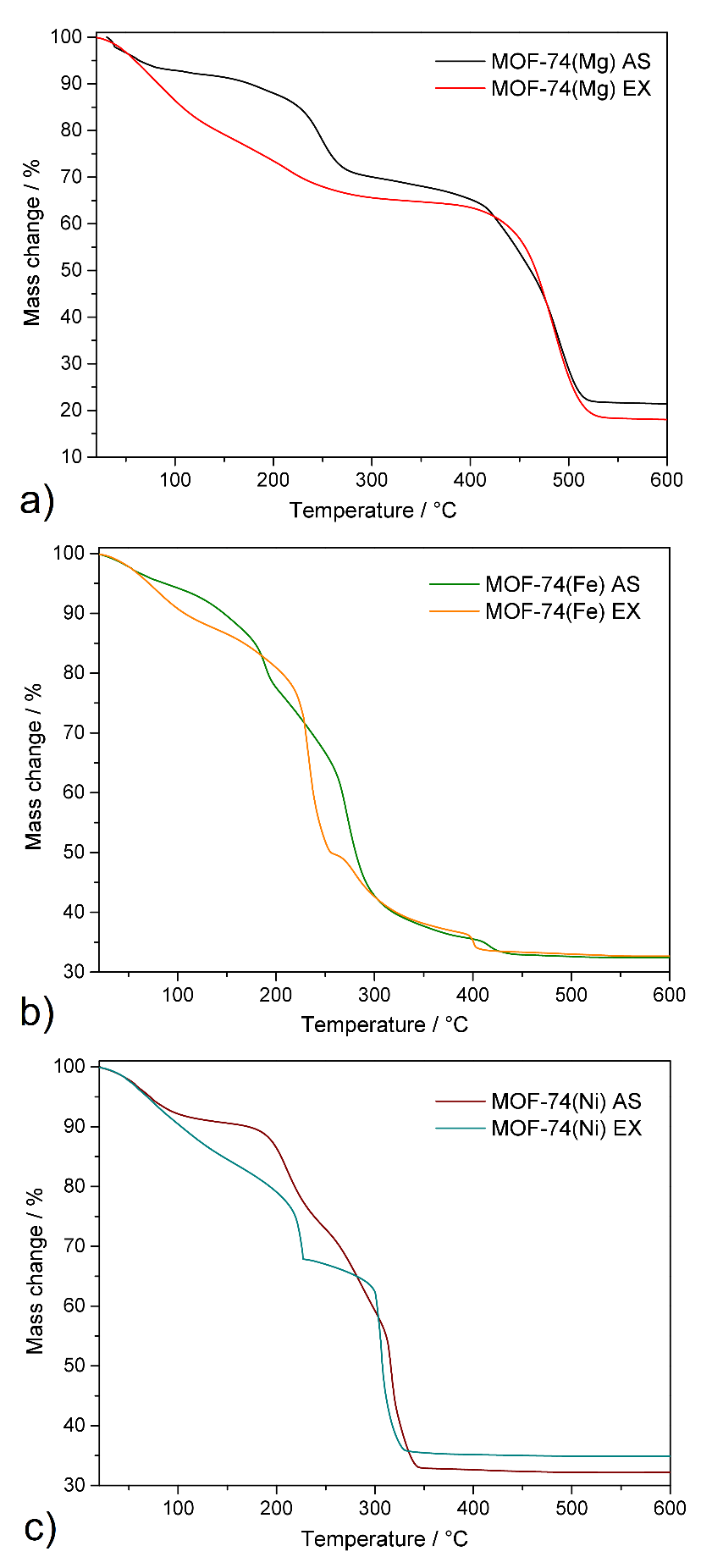


**Fig. S2** Thermogravimetric curves of as-synthesized (AS) and methanol-exchanged (EX) a) MOF-74(Mg), b) MOF-74(Fe) and c) MOF-74(Ni) samples measured in air.


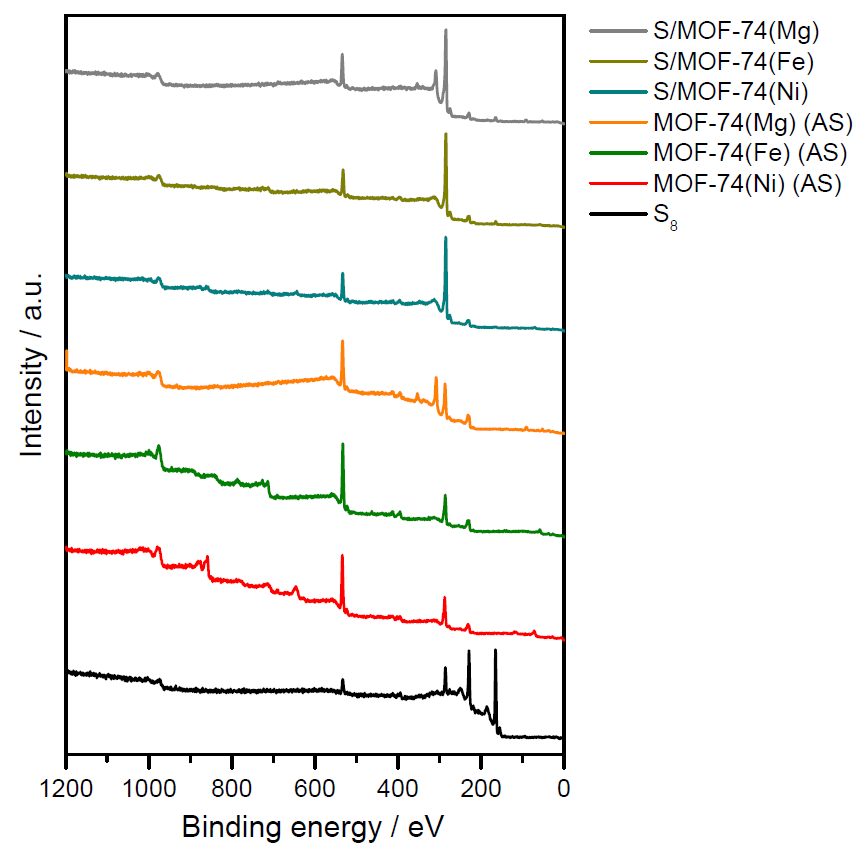


**Fig. S3** The XPS survey of octasulfur, S/MOF-74(Fe), S/MOF-74(Mg), S/MOF-74(Ni) and as-synthesized materials (AS).


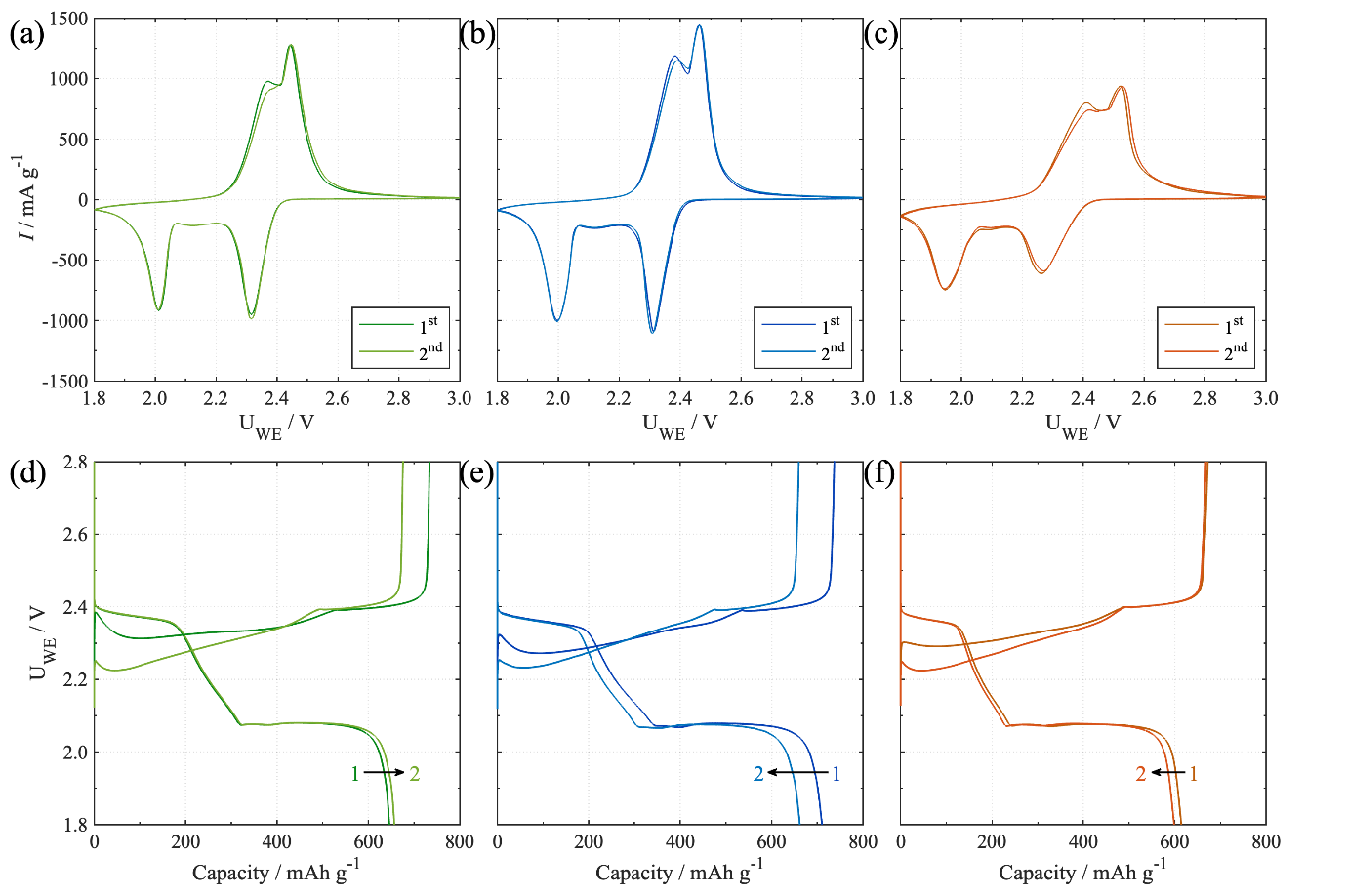


**Fig. S4** The first two cycles of CV a cycling at 0.2 C for the samples S/MOF-74(Mg) (a, d), S/MOF-74(Fe) (b, e), and S/MOF-74(Ni) (c, f).


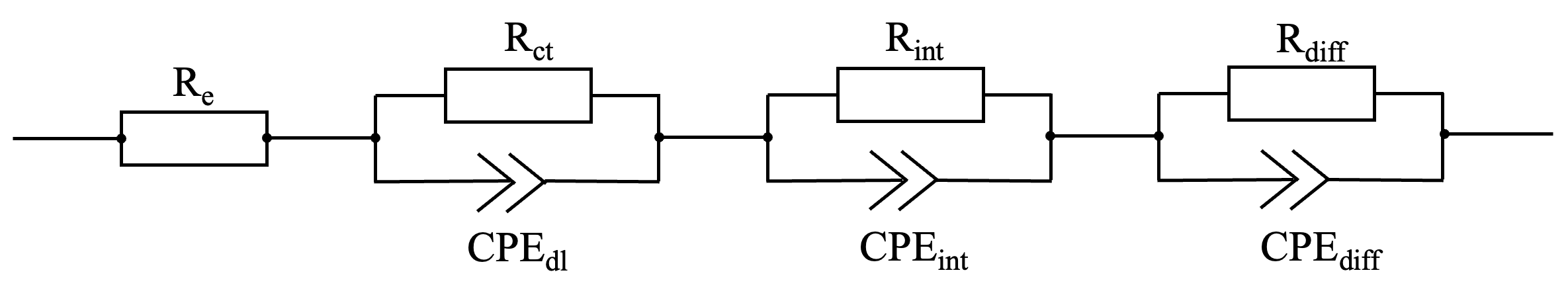


**Fig. S5** The applied equivalent circuit for fitting of EIS spectra.


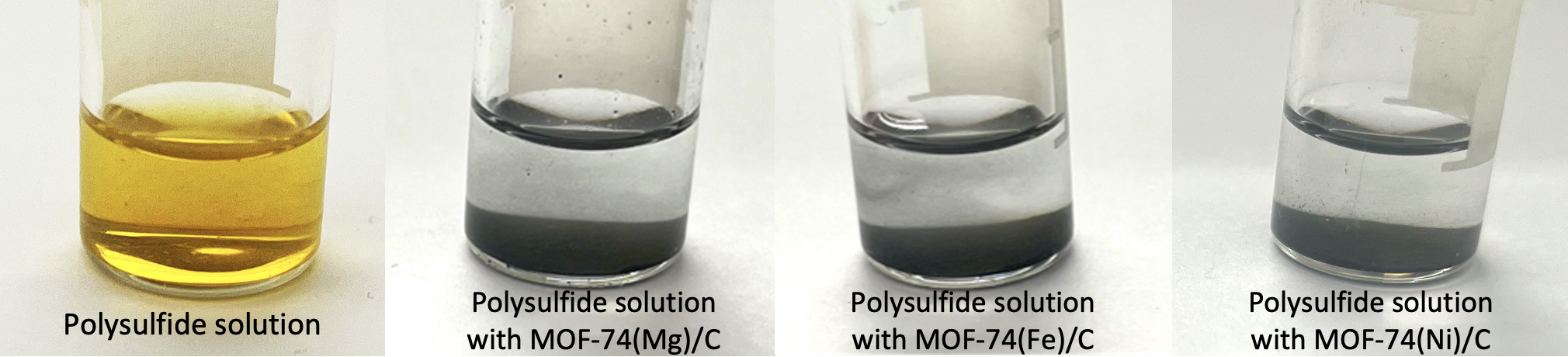


**Fig. S6** Li_2_S_6_ adsorption test on MOF-74(M)/C composites in DOL/DME mixture after 1 h.

**TABLES**

**Table S1** Empirical formulas, proposed molecular compositions, and comparison of calculated and experimental elemental analysis values (C, H, N) for MOF-74 materials with different metal centers (Fe, Ni, Mg) in as-synthesized (AS), methanol-exchanged (EX), and thermally activated forms.

| **Sample** | **Empirical formula** | **Formula** | **C (clcd)**  **%** | **H (clcd)**  **%** | **N (clcd)**  **%** | **C (exp)**  **%** | **H (exp)**  **%** | **N (exp)**  **%** |
| --- | --- | --- | --- | --- | --- | --- | --- | --- |
| MOF-74(Fe) (AS) | C_14_H_22_N_2_O_11_Fe_2_ | Fe_2_(L)·2DMF·3H_2_O | 33.23 | 4.38 | 5.54 | 33.61 | 4.47 | 5.37 |
| MOF-74(Fe) (EX) | C_13.5_H_24_O_11.5_Fe_2_ | Fe_2_(L)·5,5MeOH | 33.64 | 5.02 | 0.00 | 33.18 | 5.15 | 0.04 |
| MOF-74(Fe) (EX) 60 | C_8_H_2_O_6_Fe_2_ | Fe_2_(L) | 31.42 | 0.66 | 0.00 | 31.65 | 0.74 | 0.00 |
| MOF-74(Fe) (EX) 200 | - | - | - | - | - | 26.55 | 0.59 | 0.00 |
| MOF-74(Fe) (EX) 250 | - | - | - | - | - | 19.31 | 0.43 | 0.00 |
| MOF-74(Ni) (AS) | C_11_H_16_NO_10,5_Ni_2_ | Ni_2_(L)·DMF·3,5H_2_O | 29.51 | 3.60 | 3.13 | 29.85 | 3.42 | 3.21 |
| MOF-74(Ni) (EX) | C_11_H_14_O_9_Ni_2_ | Ni_2_(L)·3MeOH | 32.41 | 3.46 | 0.00 | 32.37 | 3.45 | 0.05 |
| MOF-74(Ni) (EX) 60 | - | - | - | - | - | 32.05 | 3.11 | 0.01 |
| MOF-74(Ni) (EX) 200 | - | - | - | - | - | 31.64 | 2.34 | 0.00 |
| MOF-74(Ni) (EX) 250 | C_8_H_2_O_6_Ni_2_ | Ni_2_(L) | 30.85 | 0.65 | 0.00 | 30.43 | 0.60 | 0.00 |
| MOF-74(Mg) (AS) | C_12,5_H_18,5_N_1,5_O_10,5_Mg_2_ | Mg_2_(L)·1,5DMF·3H_2_O | 36.94 | 4.59 | 5.17 | 37.16 | 4.81 | 5.25 |
| MOF-74(Mg) (EX) | C_11_H_14_O_9_Mg_2_ | Mg_2_(L)·3MeOH | 38.99 | 4.16 | 0.00 | 39.17 | 4.09 | 0.00 |
| MOF-74(Mg) (EX) 60 | - | - | - | - | - | 39.39 | 3.66 | 0.00 |
| MOF-74(Mg) (EX) 200 | C_8_H_2_O_6_Mg_2_ | Mg_2_(L) | 39.59 | 0.83 | 0.00 | 39.83 | 0.86 | 0.00 |
| MOF-74(Mg) (EX) 250 | C_8_H_2_O_6_Mg_2_ | Mg_2_(L) | 39.59 | 0.83 | 0.00 | 39.53 | 0.82 | 0.00 |

L – 2,5-dihydroxyterephthalate

**Table S2** Experimental elemental analysis values of C, H, N and S (in %) for MOF-74 electrode materials.

| **Sample** | **C (exp) %** | **H (exp) %** | **N (exp) %** | **S (exp) %** |
| --- | --- | --- | --- | --- |
| S/MOF-74(Mg) | 24.69 | 0.44 | 0.00 | 58.64 |
| S/MOF-74(Fe) | 23.46 | 0.41 | 0.00 | 60.88 |
| S/MOF-74(Ni) | 23.38 | 0.42 | 0.00 | 59.57 |

**Data availability statement**

The datasets used and/or analysed during the current study available from the corresponding author on reasonable request.
